# Supplementary material for: Taxed and untaxed beverage intake by South African young adults after a national sugar-sweetened beverage tax: A before-and-after study
Source: PLoS Med. 2021 May 25;18(5):e1003574. doi: 10.1371/journal.pmed.1003574 (PMC8148332; doi:10.1371/journal.pmed.1003574)
Supplement: S1 Protocol — (DOCX) [file pmed.1003574.s002.docx]

***A multimethod approach to evaluating a threshold-based sugar-sweetened
beverage tax in South Africa***

Michael Essman

**Submitted to:**

Lindsey Smith Taillie (Committee Chair)

Barry M. Popkin
Linda Adair

Francesca Dillman Carpentier

Audrey Pettifor

***Department of Nutrition
Gillings School of Global Public Health
University of North Carolina at Chapel Hill***

1. **Specific aims**

Consumption of sugar-sweetened beverages (SSBs) has rapidly increased globally^1^. SSBs are linked to increased risk of obesity^2,3^, diabetes^4^ and cardiometabolic disease^5^. Building upon evidence that national SSB taxes reduced SSB purchases in Mexico^6^ and Chile^7^ in 2014, over ten more countries have implemented an SSB tax in the last three years^8^. **However, previous research on SSB taxes has been limited because of a lack of dietary intake data. Measuring changes in dietary intake before and after the tax is critical, because the amount and quality of calories consumed is a key step on the path to obesity prevention.** Moreover, few studies have examined individual-level factors that may modify the effects of the tax such as knowledge of the health harms of SSBs. Although low knowledge about the health harms of SSBs is associated with increased SSB intake^9^, it is unknown whether changes in knowledge may modify the effects of SSB taxes. Finally, mass media framings can affect public health through second-level agenda setting, whereby media sources select which topics garner the most attention and how they should be understood^10,11^. This selective coverage can affect whether policymakers are likely to implement, maintain, strengthen or weaken the design of a policy, or add new policies. For the public, the media can influence their knowledge about SSBs and perceptions of risk, as well as their awareness and acceptance of the tax, which could influence intake. **An understanding of how media frames SSB taxes is important for contextualizing and understanding changes in SSB knowledge, perceptions, and intake before and after the tax**, and these results can help inform implementation of future policies, such as mass media campaigns to increase knowledge and awareness, that can help maximize the effectiveness of the tax.

South Africa, with one of the highest SSB consumption rates in Africa and a growing burden of type II diabetes^12^, is the first sub-Saharan African country to institute a sugary beverage tax, implemented in April 2018. They have introduced a novel tax structure that taxes each additional gram of sugar above a 4g/100 ml threshold^13^. Such an approach has not been tried nor tested anywhere. **The purpose of this proposal is to examine changes in SSB intake, SSB knowledge, and media coverage that occur in response to this SSB tax, which could serve as a model for future SSB tax evaluations.** Our specific aims are to:

**Aim 1: Estimate the pre-post changes in SSB dietary intake in South Africa.** To evaluate the South African 4g/100 ml threshold SSB tax policy, we will analyze 24-hour dietary recalls and beverage frequency intake data from the Langa 18-39y Survey of young township adults (n=2383) from Cape Town, South Africa. These data were collected at baseline (March 2018, 1 month prior to tax implementation), 6 months after, and 12 months after tax implementation. *Based on results from Mexico and Chile evaluations,* *we hypothesize that* *taxed* *SSB consumption by 18-39 year-olds living in Langa will decrease by 6% at 12 months.*

**Aim 2:** **Examine changes in tax awareness, SSB knowledge, and SSB risk perception, and determine whether these variables modify the relationship between SSB consumption and time since tax implementation.** We have collected data on participants’ awareness of the tax, knowledge about SSBs, and risk perceptions for developing obesity, diabetes, and high blood pressure as a consequence of high SSB intake. These data were collected at baseline, 6 months after, and 12 months after tax implementation. We will test whether there were changes in tax awareness, SSB knowledge, or SSB risk perception. We will then test whether there is an interaction between time and awareness, knowledge, and perceptions on SSB intake. *Our hypothesis is there will be a significant interaction between time of data collection and knowledge, such that a higher knowledge about health risks will be associated with reductions in SSB intake, and this relationship will be stronger after the tax. We expect to see similar effect modification for the other two modifiers.*

**Aim 3: Examine framing of newsprint articles related to the South African SSB tax, whether framing changes over time, and whether framing is associated with proposed causes and solutions for excessive SSB intake.** To understand public discourse around the SSB tax implementation, we will perform a quantitative media content analysis of online newspapers in South Africa for six months preceding and following the SSB tax implementation. We will examine the media response to the tax according to the framing of the relationship between SSBs and health outcomes, assignment of responsibility (i.e. individual or industry), and proposed solutions (individual behavior or policy). We will also examine whether media framings change over time. *We hypothesize that framing excessive SSB consumption as a public health problem is associated with positive messaging and tax support, and framing SSB consumption as an individual problem is associated with arguments against SSB taxation.*

1. **Significance**

**Why study the impact of SSB taxes?**

Consumption of SSBs is increasing globally^1^, and is significantly associated with increased risk of obesity^14^, diabetes^4^, and cardiovascular disease^15^. In 2010, the World Health Organization issued a call for national governments to help their citizens reduce added sugar consumption to less than 10% of daily energy intake^16^. SSB taxes are one way to reduce sugar intake in the food supply because economic disincentives can reduce purchases^17^. Recent systematic reviews have found that SSB taxes are effective in reducing SSB purchases, but their role in reducing population obesity remains unclear^18,19^. In theory, reducing SSB consumption could reduce population obesity and associated chronic diseases in the long term^20^ due to evidence that SSB demand is elastic, meaning that changes in consumption are closely proportional to changes in price^21–23^. In practice, most evidence for the health benefits of SSB taxes comes from simulations, which suggest that a price increase on SSBs could avert thousands of cases of diabetes in Mexico^24,25^, the United Kingdom^26^, Australia^27^, India^28^, Germany^29^, and South Africa^30^. SSB taxes may also reduce health care costs as they can reduce the population burden of expensive, long-term chronic diseases^31,32^. **Thus, there are large potential economic and health benefits of SSB taxes, but the mechanisms through which large scale policies may affect behavior are understudied**^31,33^**.** Showing that SSB taxes are linked to decreased sugar intake would provide strong evidence that large potential economic and health benefits are possible.

**Why is this study urgent?**

There is an urgent need for governments to make progress on population obesity prevention and reduction due to the high economic and health burden of obesity and associated non-communicable diseases (NCDs)^34^. In 2016, over 1.9 billion adults were overweight or obese, and obesity is associated with a greater chronic disease burden than underweight^35^. Since 2018, the United Kingdom implemented a threshold based tax^36^, Colombia introduced an SSB tax, and Peru modified their SSB tax rate^37,38^. **Evaluations of sugar-tiered tax rates are urgently needed to inform policymakers**. This study will improve upon SSB tax evaluations by incorporating a wider array of data that not only measures changes in actual SSB consumption before and after the tax, but also investigating pre-post measures of other modifiers of dietary choices, including knowledge and attitudes about the health effects of SSBs. Obesity is a complex, multifactorial disease, which will require comprehensive policy measures to make progress^39^. Thus, it is imperative that national-level policy analyses not only examine changes in intake before and after the policy, but also understand some of the key modifiers of behavior change. This comprehensive approach is needed to better understand the multiple pathways through which health taxes affect diets and weight outcomes. It is crucial to identify the key modifiers of behavior change to inform and improve future policies.

**What’s missing in the SSB tax literature?**

We have an opportunity to address gaps in the scientific literature by focusing on a new tax policy in South Africa that builds upon the methods used in previous studies. Previous evaluations have been used to demonstrate that these taxes reduce SSB purchases, but the ability to track changes in diets after the tax is limited to household purchase data, large aggregate purchase data, or only US based studies utilizing biased measures of dietary intake, the limitations of which are outlined below. Much less is known about SSB intake, which is critical, since it is changes in actual intake which will lead to potential changes in weight.

There have been two previous evaluations of SSBs taxes in the United States using dietary intake data, one in Philadelphia^40^ and one in Berkeley, CA^41,42^. The evaluation of the Philadelphia tax estimated both SSB consumption volume and consumption frequency, but food (and beverage) frequency questionnaires are less accurate (subject to greater bias) than 24h recalls^43^. However, when used, these problems can be improved by an internal calibration study in a subsample of the study population^44^, which was not done. One of the strengths of frequency questionnaires is they are better at estimating usual intake than 24-h recalls, but 24-h recall are the preferred method for assessing post-intervention changes in mean dietary intake in a population due to their greater accuracy^44^.

The evaluation of the Berkeley SSB tax used a BFQ that only queried SSB consumption frequency but not intake volume. Estimating caloric intakes using only frequency measures (e.g. reduction of 0.5 servings per day) would require potentially invalid assumptions about mean serving size, particularly if there are high consumers in the population who consume large amounts per consumption event. Our study includes a BFQ that also asks for amounts, which is crucial for a high consuming population and requires fewer assumptions from the researchers regarding the usual portion size, and the addition of a 24h recall allows for more accurate measure of population mean intake than a BFQ alone.

Another severe limitation of the Berkeley and Philadelphia SSB tax evaluations using dietary data was the fact that dietary intakes are not linked to food composition tables, which are necessary to calculate nutrient intakes and total calories from 24-h recalls and FFQs. As a result, the study by Zhong and colleagues^40^ is able to estimate changes in beverage volume consumption, but is unable to estimate changes in calories from SSBs, which is crucially important as changes in energy intake are central to weight loss. One of our study’s key innovations will be to develop our own food composition tables that are appropriate for the South African context, which will be then linked with the dietary assessment instruments to better estimate changes in nutrient intakes from SSBs before and after the tax.

To summarize, diet has been rarely measured in SSB tax evaluations, and so far, has only been measured in studies based in the United States. The studies that do exist mostly consist of beverage frequency questionnaires, which are a poor estimator of absolute mean intake in a population^44^. Furthermore, few studies have examined the impact of SSB taxes in largely low-income communities. This is an important addition because two thirds of cardiovascular deaths occur in low and middle income countries, and within those countries it is the lowest income communities that have the highest risk^46^. It is therefore essential to understand how low income communities, particularly those at highest risk for diet-related NCDs, are affected by SSB taxes. **Our study seeks to fill these gaps and improve upon the SSB tax evaluations to date by examining the changes in SSB consumption before and after an SSB tax using a repeated cross-sectional study of households in the Langa township of South Africa, using dietary intake data obtained from both 24h recalls and beverage frequency questionnaires.**

**Tax Structure Impact**

As more SSB taxes are implemented every year^8^, there is a growing need for evaluations to identify which tax structure is most effective for reducing added sugar consumption. Volume-based taxes are the most common^19^ approach and may be more efficient for raising revenues to apply toward health promotion subsidies^47^, but taxes based on sugar concentration may lead to greater impact on health outcomes by promoting both product reformulation as well as reduced purchases, thereby reducing the harm from excessive added sugar^48^. A tax on sugar content can incentivize industry reformulation because manufacturers can reduce the tax burden on their products by reducing their sugar content, not just by reducing sugar content below 4g/100mL but by reducing any additional sugar above this threshold (i.e. reducing sugar from 10g/100mL to 9g/mL)^49^. More data are needed to determine the tradeoffs between tax structures. The South African tax is the first of its kind, which applies a fixed 2.1 cent tax rate for every gram of sugar (both intrinsic and added) above a 4g/100 ml threshold^13^. Early calculations suggest that the average tax rate is approximately 10%. It is important to understand this novel tax structure in the context of other SSB tax evaluations because rather than having a uniform tax on volume, the 4g/100 ml threshold creates a target for industry reformulation of SSB products. A similar threshold-based multi-tiered SSB levy has been passed in the United Kingdom, but the South African tax structure is potentially even stronger as each additional gram per 100mL imposes a greater tax.

**Behavior Change is Complex and Likely Related to More than SSB Price Changes**

In addition to price sensitivity, there may be individual-level factors that influence consumer responses to SSB taxes. For example, a tax policy may have a signaling effect, meaning that awareness of the policy may increase the odds of reducing SSB consumption compared to those who are not aware of the SSB tax policy^50^. **Thus, the effect of SSB policies can be modified by factors beyond consumer’s reactions to price changes.** In Mexico, SSB consumption decreased in response to the tax to an even greater extent than economic models first predicted^6,51^. Some of this increase may be attributed to vocal and organized health advocacy campaigns that increased awareness and public acceptance^52^. Additionally, health knowledge about SSBs is associated with willingness to decrease SSB consumption^9^.

This will be the first study to investigate whether factors such as SSB knowledge may modify consumer response to the tax by changing over time (Aim 2). SSB taxes are broad tools that have been demonstrated to affect SSB purchases, but the individual characteristics which may modify responses to national policies are poorly understood. We have collected data on potential modifiers including awareness of the tax, knowledge of what beverages can be identified as SSBs, and knowledge about the risk of developing obesity and non-communicable diseases (NCDs) as a result of excessive SSB consumption. The effect of these changing potential modifiers will be estimated to understand key variables that may modify the effects of SSB taxes on consumption. This is a crucial addition to a SSB tax evaluation because we may identify potential modifiers that can be targeted by future policies to improve the effectiveness of a national SSB tax.

**Media Response Impact**

In the context of obesity prevention policy, SSB taxes are specifically designed to affect pricing and individual consumption decisions, but there may be broader societal factors that are also influenced by national level SSB taxes, which then in turn affect SSB consumption. Media representations of SSBs taxes shape public perceptions about their purpose^53^, and resulting changes in awareness of the tax and understanding of the harms of SSBs can ultimately determine whether SSB taxes will be accepted by the public^33,54^. One mode through which media affects health is second-level agenda setting, whereby media sources not only select which topics garner the most attention, but also suggest the ways in which those topics should be understood^10^. By defining a social problem and the dimensions along which it should be understood, the media can influence how both policymakers and the general public approach solutions^11,55,56^. For example, studies of agenda setting have found a strong influence of South Africa's mainstream news media in shaping the discourse about HIV/AIDS due to an influential role in politics after apartheid^57^. However, **no other studies exist that examine the effect of media on discourse about obesity in South Africa.**

The frequency of media coverage can influence the topic salience^58^, and urgency for policy action may vary with changing media coverage^55,59^. A recent systematic review of SSB tax implementation found that the framing of the SSB tax policy was crucial to whether the policy succeeded or failed in being passed and implemented^33^. Framing obesity as a disease/phenomena due to environmental factors rather than due to individual choice may improve public acceptance of government intervention and accelerate the implementation of SSB taxes^53,60,61^. Analysis of the media coverage of a SSB tax in the United Kingdom found increasing coverage of the SSB tax leading up to its implementation, a surge in opposing articles against the SSB tax, and an association between characterizing overconsumption of SSBs as an industry-driven problem and needing governmental policy solutions^62^. In summary, media framings can affect both policymakers’ decisions related to SSB tax legislation and consumers’ decisions to purchase and consume SSBs. Following the example of previous studies^11,60^, we will examine how the South African news media has framed the problem of SSB consumption, which has implications for whether taxation is an appropriate policy response that will be publicly supported.

This media content analysis will add to a growing body of literature examining the media environment related to reducing SSB consumption through taxes or other regulations^53,55,62,63^. Analyzing the framings used to debate public health policies is important because framings reflect the strategies that key stakeholders use to affect public health debates and decisions^55^. These studies can be useful to identify the conditions under which public health policies may be more or less likely to succeed. **Studies that investigate media debates on NCD risk and policy are important for developing a more nuanced understanding of the complex ways in which media representations of unhealthy commodity industries are shaped by, and contribute to shaping, public, corporate and political discourse. These analyses can provide insights into how to frame effective public health messages and counter frames that undermine public health goals.**

For our study, if the problem of high obesity in a population is discussed as a failure of individual choices, then the solutions such as education or individual level interventions will be preferred. However, previous evidence suggests that framing public health problems as being caused by systems level factors rather than individual level factors improves their susceptibility to policy solutions^11,60^. **The key consequence of this is that individualized frames suggest solutions will require individual level solutions whereas systemic frames suggest that governmental action will be required for progress.** Analysis of media representations of the tax will allow us to detect the framings that may make SSB tax acceptance more likely and to better understand the framings that are employed to either support or challenge the legitimacy of SSB tax policies (Aim 3). Including a media content analysis in our study will allow us to better understand the changing media landscape in which these changes in SSB consumption are occurring. Our multifaceted approach using multiple data sources and analysis methods is the novel contribution to methods in this field.

**Why this study population in South Africa?**

Understanding the effects of the SSB tax in South Africa is important for several reasons. First, SSB consumption in South Africa is one of the highest in Africa and continues to increase, carrying with it an increasing burden of obesity and NCDs^12^. Second, diabetes is the greatest killer of women in South Africa, and 68% of women are overweight or obese, and approximately 20% have severe obesity (BMI ≥35)^12^. This is the highest obesity rate in sub-Saharan Africa. Therefore, unless it is halted, the growing SSB consumption is likely to increase the burden of obesity and chronic disease in the future^64^. Lastly, South Africa is also the first sub-Saharan African country to implement an SSB tax, which could serve as a guide to other African communities where overweight and diet-related NCDs are rapidly increasing.

This study population in the Langa township of South Africa, selected by our collaborators at the University of the Western Cape (UWC), is an ideal study population for evaluating the changes in SSB consumption before and after the SSB tax for several reasons. First, it is the oldest settlement area and a stable community that can be followed over time. Second, it contains a large number of individuals who are heavy consumers of SSBs and are at high risk of associated chronic disease. The predominant age group within the community is 17-35 years, the highest consumers of SSBs, and the population is largely black African^65^, the group at greatest risk of chronic disease associated with poor diet^12^. Finally, this is a low income settlement with high unemployment, meaning this population is far less likely to be diagnosed and treated for sugar-related NCDs, **making primary prevention an even higher priority.**

**Why do we expect South Africa to follow a similar pattern to other countries for which we have conducted SSB tax evaluations?**

We expect to see a change in SSB consumption as a result of the recent SSB tax in South Africa based on the availability of the price elasticity of demand for SSBs in South Africa. Price elasticity is a measure of how consumers respond to price changes for specific products. If consumers are price sensitive, then they will change their purchases in response to price changes. If consumers are price insensitive, then they may not change their purchases after price changes. In South Africa, the price elasticity of demand for SSBs in South Africa is -1.18 for carbonated soft drinks and -1.17 for concentrates^66^. For comparison, the price elasticity of demand for soft drinks in Mexico was recently determined to be -1.16^22^. Additionally, there was a greater reduction in taxed SSBs among low-income populations in Mexico^6^. This suggests that the **one-year** **expected change in SSB consumption in response to the tax in South Africa should be of similar magnitude to the one-year change in Mexico (6%).**

1. **Innovation**

Our SSB tax evaluation attempts to measure post-tax changes in SSB consumption and to understand the multiple pathways through which a SSB tax may operate, including the media response and the changes in knowledge and beliefs about SSBs and their relationship to health. **This study is innovative because no prior study has integrated all of these data to get a more comprehensive understanding of *if* and *why* the tax affects SSB consumption, which is in turn linked to obesity and other diet-related NCDs.** Our key innovations include 1) collecting diet data on a large sample of high-consuming young adults to better understand the effects of the tax on actual consumption behavior instead of only household level purchases;
2) developing an up to date food composition table to link with these dietary data to estimate both changes in volume and nutrient consumption after an SSB tax; and 3) assessing the changes in knowledge and attitude that follow the SSB tax implementation and their associations with intake. The central innovation of this study is the improved dietary assessment methods and dietary data management plan that allow for a more accurate assessment of SSB intake compared to previous evaluations. One of our innovations is also conceptual: no SSB tax evaluation to date has attempted to incorporate potential modifiers of the relationship between SSB taxes and SSB consumption.

1. **Approach**

**Preliminary Studies**

South Africa is an important country to have a SSB tax evaluation due to rising national level SSB consumption trends, rising chronic disease, and being the first African country to implement an SSB tax. Using data from Euromonitor International^67^, we found that SSB sales are rising rapidly across the Africa and Middle East region (Figure 1), and one of the highest per capita consumption rates of SSBs is found in South Africa^12^. While SSB consumption in other high- and middle-income countries is staying constant (Mexico) or decreasing (USA and Chile) in recent years, our data show that SSB consumption in South Africa is rapidly rising and is approaching the levels of other high SSB consumption countries that have implemented SSB taxes. Therefore, there is an urgent need to understand SSB consumption trends and how they may change in response to the new South African SSB tax.

**Figure 1.** Trends in total SSB sales in the USA, Mexico, Chile, South Africa, and the Africa & Middle East Region from 2003-2017. SSBs include categories of regular cola carbonates, non-cola carbonates, liquid concentrates, powder concentrates, juice drinks of <99% juice, sport and energy drinks, and ready-to-drink coffee and tea. All data were derived from the Passport Global Market of Euromonitor International^67^. SSB, sugar-sweetened beverages.

We have conducted descriptive analyses of the baseline demographic and dietary intake data for our study population. Table 1 below displays the baseline SSB intake according to the beverage frequency questionnaire. These data suggest that per capita SSB intake in Langa is much higher than the average per capita SSB intake in South Africa estimated from Euromonitor data in Figure 1.

**Table 1. Mean unadjusted daily volume (ml) of beverage consumption from beverage frequency questionnaire for participants in Langa 18-39y Survey (n=2383), collected at baseline (prior to tax implementation). Data are presented as consumption per capita and per consumer.**

|  | **mL per capita** |  | **% Consumer** | **mL per consumer** |  |
| --- | --- | --- | --- | --- | --- |
| **SSBs** | Mean | SD |  | Mean | SD |
| Water (flavored) | 27 | 114 | 27 | 104 | 207 |
| Concentrates/Cordials | 280 | 460 | 78 | 371 | 496 |
| Sodas (regular) | 403 | 689 | 91 | 449 | 713 |
| Sports and Energy Drinks | 105 | 215 | 62 | 187 | 256 |
| Coffee/tea (sweetened) | 162 | 281 | 65 | 261 | 319 |
| Other* | 40 | 144 | 40 | 120 | 190 |
| Total SSB | 1054 | 1071 | 98 | 1075 | 1071 |
| **Non SSBs** | Mean | SD |  | Mean | SD |
| Water (tap and bottled) | 1289 | 1484 | 99 | 1260 | 1339 |
| Diet sodas | 7 | 45 | 8 | 103 | 139 |
| 100% fruit juices | 58 | 211 | 53 | 115 | 285 |
| Coffee and tea (unsweetened) | 17 | 208 | 5 | 393 | 922 |
| Milk (unsweetened) | 143 | 244 | 71 | 213 | 271 |
| Total Non SSB | 1515 | 1598 | 99 | 1537 | 1599 |
| **Alcohol** | 160 | 1186 | 36 | 446 | 1950 |
| **Total beverages** | 2729 | 2487 | 100 | 2729 | 2487 |

*Beverages include powdered drinks, sweetened milk, and sweetened iced tea.

**Methods**

*Overview*

The overall purpose of this project is to (1) estimate the changes in taxed and untaxed beverage consumption that follow a novel SSB tax implementation; (2) examine SSB knowledge, SSB risk perception, and tax awareness are potential modifiers of the relationship between SSB taxes and SSB consumption; and (3) quantitatively examine the media response to the South African SSB tax. There is a strong empirical basis for conducting this study due to the evidence that SSB taxes have significant effects on SSB consumption^6,7,19,45^, that low knowledge about the health harms of SSBs is associated with increased SSB intake among adults^9^, and that public acceptance of policies are shaped by media framings^53,56,68,69^ and awareness^50^ of the policies. This study is premised upon the conceptual model presented in Figure 4, which shows how the effect of SSB taxes on SSB consumption may be modified by consumers’ knowledge about SSBs and understanding of their relationship with health outcomes as well as awareness of the SSB tax. Additionally, the discussion surrounding new SSB tax implementation generates a media response, the effect of which is partially mediated through changes in consumer knowledge and attitudes as well as other possible mechanisms whereby public discussion of taxes may affect SSB consumption. Thus, both baseline consumer knowledge as well as media influences on consumer knowledge affect SSB consumption. We proceed to outline the approach for each of our aims below.

**
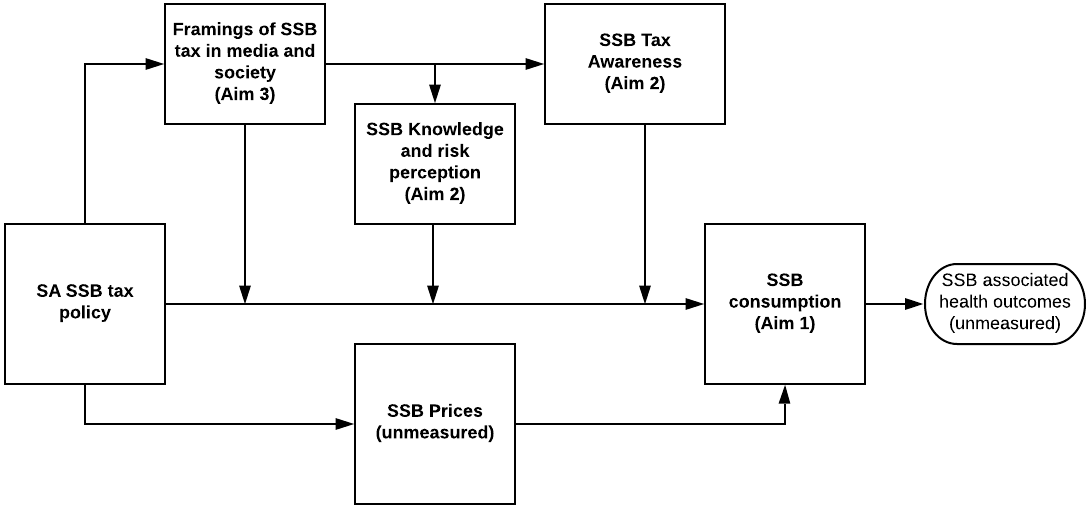
**

**Figure 4.** Conceptual Model of the relationship between SSB tax policy, its effect on the media, and the downstream effects on consumer knowledge and tax awareness. These variables are theorized to modify the effect of the relationship between time since taxation and SSB consumption.

*Key databases*

**Langa 18-39y Survey:** Dietary data will be analyzed from the Langa 18-39y Survey of young township adults (n=2383 households), collected by the University of the Western Cape in Cape Town, South Africa. The study population is comprised of adults aged 18-39y living in the Langa township of South Africa. Consent was obtained from 2,383 households to participate in the study from the 2,621 that were requested in the door-to-door recruitment process in the township (90.9% acceptance rate). The Langa township was selected due its proximity to the University of the Western Cape, the stability of the community that could be followed over time, and because it contains a large number of individuals who are heavy consumers of SSBs. Participants resided in a low-income area and received a financial incentive of R30 (USD$2.19) for Shoprite, the supermarket in Langa, after participating.

Data collection began in February 2018, two months before the SSB tax implementation, in order to collect baseline dietary intake data before the SSB tax was implemented. Data are collected at six months and twelve months following the SSB tax implementation to measure changes in the diet of the population. The study instruments, outlined below in Measures and Data Collection, include the 24-hour dietary recall assessment, a beverage frequency questionnaire, and questions related to participant knowledge about SSBs and their associations with increased risk of NCDs.

Participants were recruited using a door-to-door sampling method of all identifiable households in Langa until the target sample size of 2,500 households was achieved. The Langa township has 17,402 households and 52,401 inhabitants (50.4% female), of which 99.1% are of Black African race. The only eligibility criteria applied during data collection was age: participants aged 18-39y were invited to participate in the survey as the investigators were specifically interested in the SSB consumption of young adults. For the baseline data collection, 90.9% of requested participants consented to participate in the study.

**Table 2. Sociodemographic information for Langa 18-39y Survey (n=2,383)
compared to South African National Sociodemographic Statistics 2017**^70^

| **Variable** | **Langa sample**  **(n=2,383)** | **South Africa 2017 (n=56,521,900)** |
| --- | --- | --- |
| **Sex** |  |  |
| Male (%) | 35% | 49% |
| Female (%) | 65% | 51% |
| **Age**  Mean (SD)  Median | 27.9 (6.0)  27.0 | --  26.3 |
| **Race (% Black African)** | 99.1% | 80.8% |
| **Male overweight/obese* (%)** | 25% | 31% |
| **Female overweight/obese* (%)** | 69% | 68% |
| *Overweight/obese defined as BMI ≥ 25. | | |

*Measures and Data Collection*

Data on dietary intake, knowledge and beliefs about SSBs were collected at each time point. For the **diet assessment**, 24-hour diet recalls were conducted by interviewers with nutrition training. Participants reported what foods and drinks were eaten, how foods and beverages were prepared and whether anything was added, and the quantity consumed. Participants were prompted to recall all foods and beverages consumed at six defined time periods throughout the day including: Breakfast consumed between waking up until 9:00, Mid-Morning consumed between 9:00-12:00, Lunch consumed between 12:00-2:00pm, Afternoon consumed between 2:00pm-5:00pm, Supper Time consumed between 5:00pm-sunset, and all other consumption after supper, before bed, and throughout the night. All consumption that occurred during these specified periods was recorded, and no distinction was made between eating occasions (e.g. snacks versus meals). Participants also reported a binary indicator of yes/no for whether this was a usual day of eating for them and if not, what was the reason that intake was unusual. For the **beverage frequency questionnaire** (for simplicity will be referred to as FFQ), participants were asked about two components of their beverage intake: the most common serving size consumed and frequency of intake. For frequency, they were asked “During the past month how often did you (personally) consume any of the following beverages” for each beverage category presented in Table 3, after which they were asked the most common volume (mL). From this survey, baseline beverage intakes from beverage categories are presented in Table 1 above.

After completion of the dietary intake assessment, participants completed a **knowledge and attitudes questionnaire**. This survey asked whether the participant could classify the beverage categories listed in Table 3 as either SSBs or non-SSBs as a demonstration of their knowledge of beverages containing added sugar (hereafter referred to as “knowledge”). Participant knowledge about SSBs was measured using a 1-3 scale for each beverage category listed in Table 3. Participants were asked whether each beverage category was (1) not sugary, (2) somewhat sugary, (3) sugary, or (4) do not know to determine the ability to identify SSBs among beverage categories. Next, after participants were given a definition of SSBs, they were asked to what degree SSB consumption increases the risk of a number of chronic diseases and risk factors including diabetes, high blood pressure, obesity, dental problems, and cancer (hereafter referred to as “risk perceptions”). The degree to which SSBs increased the risk of these conditions could be answered as (1) Not at all, (2) A little, (3) Somewhat, (4) A lot, or (5) Not sure. To assess perception of the tax, participants were asked (1) whether they were aware of the SSB tax (yes/no) and (2) whether they supported the taxation of unhealthy foods and beverages, answerable with strongly oppose, oppose, agree, or strongly agree. Finally, participants were asked whether they intended to reduce their consumption of any of the categories in Table 3 or their SSB consumption overall (“intentions”). Only one diet assessment and one knowledge questionnaire were completed for a single individual within a given household. If there was more than one person in the household, then the older individual (usually mother or grandmother) completed the household level questions. There were no households within which multiple individuals completed these assessments. At both 6 and 12 month data collection periods, participants were asked whether they had been previously surveyed, and if so, at which time period.

In addition to these individual-level data outlined above, another questionnaire was completed using household-level data. Socioeconomic status was assessed according to the SAARF Living Standards Measure (LSM), developed by the South African Advertising Research Foundation (SAARF). The LSM has 10 levels for the South African population, and the score development has been described elsewhere^71^. Our collaborator states that the majority of our population is LSM 5 or lower, which correspond with the bottom 45% of the country^72^, but we still need to verify the distribution of the LSM in our data. The measures necessary for the LSM were collected at baseline, 6 months, and 12 months.

Data: Beverage classification system

To group beverages, we will use a system previously developed to classify beverages in the US^73^ and Mexico^74^, adapted to the South African context. This allows us to compare beverage categories across countries while also ensuring that the beverage groups reflect South African’s typical beverage consumption patterns and that nutritionally similar beverages are grouped together. We will classify beverages into ten broad categories: 1) Water (bottled or tap) and flavored water (either sweetened or unsweetened); 2) cordials and concentrates 3) nectars; 4) energy and sport drinks; 5) regular sugar sodas or soft drinks; 6) diet sodas; 7) 100% fruit juice drinks; 8) coffee and tea; 9) milk and dairy products; and 10) powdered drinks. Table 3 contains descriptions of each beverage analysis category. Each of these categories will be further designated as either taxed or untaxed according to whether they contain added sugars above (taxed) or below (untaxed) the 4g/100ml threshold. Because the amount of tax increases with each gram of sugar, we may also consider further dividing taxed beverages into two categories: taxed (>= 4g/100mL and <8 g/100 mL) and high-taxed (>=8 g/100 ml).

**Table 3. Beverage groups used for classification**

| **Beverage Group Name** | **Beverage group description and examples** |
| --- | --- |
| ***Taxable*** | Beverages that are not exempted, and therefore potentially taxable |
| Flavored water | Still or sparkling water with added flavoring or sweeteners |
| Cordials/concentrates | (e.g. Oros, Fusion, Wild Island) |
| Nectars | Nectars or canned juices that contain fruit (e.g. Tropica, Cabana, Halls, Elvin, Dalys, Take5) |
| Sports and Energy drinks | Energy (e.g Score, Red Bull, Monster) and Sports (e.g. Energade, Powerade, Lucozade) |
| Regular soda or soft drinks | (e.g. Coca Cola, Sprite, Fanta, Stoney Ginger Beer, Cream Soda, Dry Lemon, Jive, Twizza, Refresh) |
| Sweetened milk and dairy products | Sweetened & flavoured: (e.g. Nesquik, Steristumpi, Yogisip) |
| Coffee / tea | Sweetened or unsweetened: (bottled or served; including cappuccino etc) Store bought or home prepared coffee and tea without added sugar |
| ***Untaxable*** |  |
| 100% fruit juice | (e.g. Liquifruit, Ceres, Appletizer) |
| Water (tap, bottled) | Still or sparkling water, without added flavoring or sugars |
| Diet soda / artificially sweetened (noncaloric) | (e.g. Coca Cola Light, Tab, Sprite Zero) |
| Unsweetened milk and dairy products | Unflavored and unsweetened: (e.g. full cream / low fat / fat free / amasi) |
| Powdered drinks | (e.g Game) |

*Linking Food Composition Tables to Beverage Categories*

Accurate food composition tables (FCTs) are necessary to calculate nutrient intakes and total calories from 24-h recalls and FFQs. As a result, inappropriate FCTs for a study population can lead to substantial error in estimations of nutrient intake^75,76^. Because policies such as taxes tend to incentive reformulation, the use of an up-to-date RCT that reflects a rapidly changing food supply is especially important for evaluating policy effects on dietary intake. One key innovation of our study is that along with UWC, we will develop up-to-date FCTs for South African beverages before and after the tax, which will be then linked with the dietary assessment instruments. This will ensure that we are able to understand which beverages are taxed/untaxed at each point in time, and therefore to estimate mean volume and calories from taxed and untaxed beverages in our population before and after the tax. The FFQ contains categories exactly as written in Table 3. Therefore, we will be able to use our updated FCT in analyses using both the 24-h recall and the FFQ. We will also be able to analyze taxable versus untaxable beverages using the FFQ, given that questions make distinctions between taxable beverages and beverages that are specifically exempted.

First, **nutrition facts panel data** was collected by UWC in February and March 2018 (the two months prior to tax implementation). Fieldworkers from UWC took photos of packaged foods in grocery stores (e.g. Shoprite, Checkers, Pick N Pay, Spar, Woolworths), which contained information including barcode, name, size, nutrition, ingredients, and preparation instructions for various beverage brands included within the beverage types listed in Table 3 below. Next, research assistants at UNC linked UWC’s NFP data to FCT beverage codes, e.g. records for various brands and flavors of regular/caloric soda would all be linked to one FCT code for regular/caloric soda. Then, programmers at UNC created an **average nutrient profile** for each **food composition table beverage code**, weighted by the household purchase data from Kantar World Panel (with beverage records with higher purchases contributing more to the nutrient profile), to better reflect the beverages purchased (and consumed) during their time period of collection. Our created food composition table (the UWC-UNC FCT) beverage codes will therefore have values for total sugar concentration, allowing us to assign to them taxation status according to South African law. **Taxation status** will be determined by a two-step process. First, we ask the question of whether the product category is taxable, as 100% fruit juice and milks are exempted^77^. Among those beverages that are taxable, those with a total sugar concentration greater than 4g/100ml are classified as taxed and those with 4g/100ml or less are untaxed. Once the food composition table beverage codes are complete and assigned taxation status, they can be **linked to the beverages reported from the two dietary assessment instruments using beverage codes**. This combination of the updated UWC-UNC FCT with diet assessments will allow us to estimate volume and energy intakes from taxed and untaxed beverage categories and subcategories in our study population.

In addition to this baseline food composition table, we will also develop a renewed FCT using nutrition facts panel data collected 12 months after the tax (March 2019) to account for potential reformulation that has occurred since the tax was implemented. Using the same baseline FCT at both time points will show behavioral change only, since we effectively assume no reformulation by keeping the same FCT. However, using the 12-month updated FCT allows us to understand how our analysis may be affected by reformulation. The approach is outlined in greater detail in the Aim 1 Analysis Plan below.

*Study Power*

This study is powered to detect a mean difference of 6% in SSB intake in our population by 12 months compared to baseline, with 80% power at an alpha level of 0.1. Baseline data from the beverage frequency questionnaire suggests that SSB consumption is 872 ml/day (SD 603); our study would require 1,913 subjects to detect a 6% reduction in SSB intake.

**Aim 1: Estimate the post-tax changes in SSB intake after implementation of the South African SSB tax policy.**

**Data and Subjects**

We will analyze 24-hour dietary recalls and beverage-related FFQ data from the Langa 18-39y Survey of young township adults (n=2383) from Cape Town, South Africa. We will estimate beverage intake at baseline, 6 months, and 12 months of data collection, with beverages categorized as taxed/untaxed according to the 4g of sugar per 100ml threshold.

**Outcomes**

Our primary outcomes are taxed beverage volume, taxed beverage calories, untaxed beverage volume, and untaxed beverage calories. Secondary outcomes will include volume and calories of taxed and untaxed beverage sub-categories (Table 3), as well as total beverages.

**Analysis Plan**

*Adjusted estimates of overall taxed and untaxed beverages*

Our first objective will be to perform a descriptive/unadjusted analysis, examining the mean and distribution (intakes for top and bottom quintiles) of taxed and untaxed beverage categories and subcategories (Table 3) at each time point, as well as the percentage of our sample that reported consuming beverages in that category.

Our next objective will be to examine the mean adjusted volume and calories per capita and per consumer at baseline, 6 months, and 12 months after the SSB tax was implemented for overall taxed and untaxed beverages. We will perform this analysis using two different approaches with respect to the FCT linkage used. For the first approach, we will analyze beverage intakes using the February/March 2018 UNC-UWC Food Composition Table linked to baseline, 6 month, and 12 month data. Pre-post beverage intake comparisons will be made using this same FCT across all three time points. As a result, changes in mean taxed SSB intake will reflect **behavioral changes only**, since we effectively assume no reformulation by keeping the same FCT. In other words, the only way taxed beverages can decrease is for consumers to consume less (as opposed to beverages shifting from being taxed to untaxed due to reformulation).

For the second approach, to account for possible effects of reformulation and the fact that the nutrient composition of the beverages may be changing over time, we will use our 2018 FCT for baseline data and then link the updated February/March 2019 FCT to the dietary intake data for 12 months. In this approach, changes in the mean volume of taxed beverage intake could be driven by changes in behavior or **changes in taxed beverage classification** (if beverages switch from one side of 4g/100ml threshold to the other). In addition to these reasons, changes in the mean calories of taxed beverages could be due to **changes to within-beverage category sugar concentration** (e.g. taxed beverages remain taxed but reduce their caloric content). We will not repeat the analysis for 6 month data because updated nutrition information was not collected at 6 months.

Linear regression models will be used to estimate taxed and untaxed beverage consumption at each time point, adjusted for the covariates listed in Table 4. We will estimate unadjusted results, adjusted results (including individual level-covariates, weekday of intake, and temperature), and finally results adjusted for these covariates plus BMI. These models will include adjustment for total energy when using 24-h recall data. Given that our FFQ cannot be used to obtain total energy intake, adjusting for BMI will allow us to potentially control for variation in total energy intake in our sample. Comparisons between baseline and 6 month and between baseline and 12 month means will be made with two-tailed t tests. We plan to use both the 24-h recall and the FFQ for these analyses. For the FFQ, we will multiply the amount reported as most commonly consumed times a frequency per day (e.g. intake amount of once per week would be multiplied by 1/7) to obtain daily intake.

**Table 4. Outcomes and Covariate measures for model adjustment**

| **Primary outcomes** |  |
| --- | --- |
| Taxed beverage volume | Taxed based on having more than 4g of sugar per 100ml |
| Taxed beverage calories |  |
| **Secondary outcomes** |  |
| Untaxed beverage volume | Untaxed based on having less than 4g of sugar per 100ml |
| Untaxed beverage calories |  |
| **Potential modifiers** |  |
| SSB knowledge | Latent variable obtained from confirmatory factor analysis (CFA), for knowledge of whether selected beverages contain added sugars. |
| SSB risk perception | Latent variable obtained from confirmatory factor analysis (CFA), for perceived risk of NCDs due to SSB consumption |
| **Covariates** |  |
| Individual-level covariates | Age (continuous, range 18-39), sex, total energy intake, BMI, smoking (yes/no), alcohol intake, socioeconomic status (ordinal categorical), disease status (diagnosis of diabetes, heart disease, high cholesterol, high blood pressure, cancer, or obesity) |
| Weekday | Diet intake reported for weekday versus weekend (binary) |
| Temperature | Obtained from South African Weather Service, to control for seasonal changes in SSB intake |
| Time period | Categorical: 0 (baseline), 1 (6 months), 2 (12 months) |

**Expected Results:** Based on results from Mexico and Chile where an SSB tax was implemented, we hypothesize that taxed SSB consumption by 18-39 year-olds living in Langa will decrease by 6% at 12 months. Based on the magnitude of change reported from previous SSB tax evaluations, we do not expect to have sufficient power to detect a statistically significant change in taxed SSB consumption by 6 months.

*Analysis of taxed/untaxed beverage subcategories*

We will repeat the above analysis for beverage subcategories (Table 3), using both approaches for linking FCT data to dietary intake data to account for potential reformulation across beverage subcategories.

*Comparison of diet assessment methods*

Our study utilizes two different methods of diet assessment, one 24-h recall and one FFQ, to measure beverage intake in our Langa population. We will perform separate analyses using data from the **24h recall** and **beverage frequency questionnaires** to estimate intakes of our key outcome variables including total beverage calories, taxable beverage calories, untaxable beverage calories, and total sugar content. We will then compare agreement between the change in mean intakes of these key outcome variables from baseline to 6 months and from baseline to 12 months for the two sources of dietary intake data. We will calculate correlation coefficients to statistically compare agreement depending on the outcome of a Shapiro-Wilk test for normality of the nutrients under consideration. If the distributions are normal, then we will use Pearson correlation coefficients. If the distributions are non-normal, then we will use the nonparametric Spearman correlation coefficients^78,79^.

Using both the 24h recalls and FFQ will provide a more complete picture of change in beverage intake, as there are different strengths and weaknesses of each dietary assessment method. Compared to food frequency questionnaires, 24h hour recalls are a more accurate measure of dietary intake, but are subject to greater within-person variation. Because this within-person variation in day to day intake is due to random error, the mean random error is expected to be zero over a population if the administration of the recall is collected across days of the week^44^. Therefore, a single administration of a 24-hour recall on the entire sample at both time points is a suitable and sufficient method for performing a pre-post intervention assessment of mean dietary intake^44^. Thus, our main outcome will be beverage intake as measured by the 24-h recall.

However, one key limitation of using 24-h recalls for capturing dietary intake is that they **may not capture episodic consumption of some beverage categories** and are subject to greater variance when only one recall is used^80,81^. In this study, because overall consumption of SSBs is high (>90% consumers), this is less of a concern for overall SSBs than it is for beverage sub-categories, which may be more likely to be episodically consumed. To address this concern, usual intake methods such as the NCI method^82,83^ or MSM^84–86^ have been developed; however, these methods require at least two 24-h recalls in a subsample of the target population, which we do not have. Therefore, instead of using usual intake methods, we plan to address this issue by using both the 24h recall and an FFQ to examine pre-post population mean intakes. Using the FFQ to examine pre-post means is advantageous because it covers a longer time period than the 24h recall and thus better reflects usual consumption.

In addition, FFQs can be used to supplement 24-h recall data to distinguish true nonconsumers from occasional nonconsumers^85^. We will examine how well the 24-h recall captures consumers compared to the FFQ by first identifying those who report any consumption of a beverage category according to the FFQ. We will then determine what percentage of true consumers are also identified as a consumer in the 24h recall.

Using regression models with a high number of non-consumers (zero value for consumption) can bias results. For beverage categories with <90% consumers, we will estimate beverage intake from 24-h recalls using a two-part model. The basic structure of the two part model is: *Total amount of beverage category consumed = [Probability to consume beverage category] * [Amount of beverage category if consumed]*. First, the two-part model uses a logistic regression model for the binary choice of the probability to consume the beverage. Second, conditional on a positive outcome, an OLS regression model with log-transformed y in the second part is used to model the beverage consumption^87^. Standard errors and confidence intervals are calculated for retransformed values using a nonparametric bootstrap from the Stata **margins** command^87^. Models are adjusted for the same covariates in both steps.

**Limitations**

*Study design*

Given that our data are cross-sectional and not longitudinal, we are not able to follow individuals over time, only measure differences in population means. Social desirability bias could affect reporting of SSB intake and cause us to underestimate SSB intake. It is also possible that after the tax, social norms may have shifted so that the effect of social desirability bias is even greater after SSBs are subject to tax, causing an overestimation of reductions in SSB intake in this population.

*Repeated individuals across data collection periods*

We are using a repeated cross-sectional study design, and it is possible that we have repeated individuals surveyed at more than one time point. We do have a variable that indicates whether participants were included in our sample at previous data collection points, but the individual identifiers were generated using a method that does not allow for individuals to be linked across time. This limitation could potentially bias our results by an unknown magnitude and direction, given that we cannot know whether repeated individuals were high or low consumers of a given beverage category.

*Accuracy of FCT beverage codes*

Although the beverage codes that we are developing for our UNC-UWC FCT are based on nutrition information of South African beverages, they still represent an average and therefore it is possible that some beverages may be misclassified as taxed or untaxed if the actual products consumed differ in their sugar content to the average sugar content calculated for the beverage category. For example, if there is brand-specific reformulation, where some soda brands reduce their sugar (to become untaxed) and others do not (remain taxed), this could lead to misclassification. The potential for misclassification of beverages is even greater for the FFQ versus the 24-h recall because the beverage categories reported are broader than the individual beverage codes contained within each beverage subcategory.

**Aim 2:** **Examine changes in tax awareness, SSB knowledge, and SSB risk perception, and determine whether these variables modify the relationship between SSB consumption and time since tax implementation.** The key goal for Aim 2 is to test whether tax awareness, knowledge, or risk perception change over time after tax implementation and whether these variables modify the relationship between SSB consumption and time since tax implementation. We will test (1) whether there are significant differences in mean SSB knowledge, risk perception and prevalence of tax awareness before and after the tax (2) whether there is a time*modifier interaction on taxed SSB intake, pooling data from baseline, 6, and 12 months. Aim 2 will be completed using the same study population as Aim 1. The rationale for this analysis is depicted in Figure 4, where SSB knowledge score modifies the relationship between SSB tax implementation and SSB consumption.

**Outcomes**

The main outcomes in this aim include the binary variable tax awareness and the latent variables SSB knowledge and SSB risk perception, which we will measure with confirmatory factor analysis.

**Confirmatory Factor Analysis Variable Construction**

Measurement tools for participants’ SSB knowledge and SSB risk perceptions are described in “Measures and Data Collection” above (p. 9). Responses to SSB knowledge and SSB risk perception questions will be used to develop two distinct composite measures for our SSB knowledge score using confirmatory factor analysis performed in MPlus.

Confirmatory factor analysis is a method for establishing the validity of using a set of observations for measuring a latent variable. A set of survey questions is typically developed because there is a theoretical variable that explains the responses to observable variables. For example, the condition of having depression might explain a person’s responses to a mood questionnaire, even if depression itself is not directly observable. One approach to measuring depression might be to sum or average the scores on depression-related questions. However, each observable survey question has considerable measurement error, the magnitude of which will be unequal for each question^88^. Such cases benefit from using statistical models like confirmatory factor analysis that can reduce this measurement error, and using multiple indicators of a construct contains more information than any survey question individually. In our case of measuring responses to SSB knowledge and risk perception questions, any single question identifying a certain is better understood as a composite measure of all the answers than using the questions individually. Also, given that our survey questions on SSB knowledge and SSB risk perception are expected to be answered similarly according to participants’ true underlying knowledge and risk perception, including all of these variables separately in a regression model would introduce problems of variable multicollinearity.

Therefore, we will conduct a confirmatory factor analysis because this statistical technique accounts for differences in measurement error between each of the questions used to measure the latent variables SSB knowledge and SSB risk perception. Preliminary results from confirmatory factor analysis using baseline data suggest that our survey questions are effectively measuring the latent variables of SSB knowledge and risk perception according to overall model fit, R squared values for each observed variable (the survey questions), and statistically significant factor loadings. Chi-square fit statistics are not appropriate for this study as it is highly influenced by sample size, with larger samples (>400 observations) making it more likely to have a statistically significant model fit^89^. Instead, we will use the Comparative Fit Index (CFI), as it is less sensitive to sample size. A value of at least 0.90 is considered a good model fit^90^, and according to results generated in MPlus using our baseline data, the CFI is 0.972 for SSB knowledge and 0.999 for risk perception, both indicating excellent model fit^90^. The Root Mean Squared Error of Approximation measures closeness of fit, and our baseline RMSEA is 0.034 for SSB knowledge and 0.014 for risk perception, both meeting the <0.05 criterion for good model fit^90,91^. We will repeat the model fit testing at each time point separately to ensure the models fit the data sufficiently well, and we will perform a series of configural invariance tests for each separate time period to test whether the same CFA is valid in each group. These tests ensure that factor loadings and intercepts are the same across groups and the responses are therefore comparable.

After the confirmatory factor analysis, we will first test whether our potential modifiers change significantly over time. This will be done by setting our latent variable—for SSB knowledge and then for risk perception—as a continuous dependent variable in a linear regression model and entering time (coded 0, 1, 2 for baseline, 6 and 12 months, respectively) as the independent variable. The coefficient for time will indicate the change in knowledge (or risk perception) for a unit change in time period, and the p value will indicate whether this result is statistically significant. For the binary variable tax awareness (yes/no), we will measure change over time using two sample t-tests, comparing the mean awareness at 6 months to baseline and 12 months to baseline.

Next, we will determine whether SSB knowledge, SSB risk perception, or tax awareness modify the relationship between time and taxed beverage consumption. Using linear regression models, we will test whether the relationship between our potential modifiers and taxed SSB consumption differs by time using interaction terms. We will test for interaction with a Wald test. We will conduct a pooled analysis using all observations from baseline, 6 months, and 12 months post-tax when testing these models, with dummy variables coded 0, 1, 2 to indicate each data collection time period. Our potential modifiers of SSB knowledge and risk perception will be kept as continuous variables because categorization results in a loss of information, and there is no substantive reason for creating arbitrary cutoffs when testing this interaction. Models will be controlled for individual level covariates listed in Table 4, weekday of interview, and temperature (to control for seasonality).

**Expected Results:** In accordance with our conceptual model, we expect to see tax awareness, SSB knowledge, and SSB risk perception to increase over time. We expect a significant interaction between time of data collection and SSB knowledge score, such that a higher knowledge about health risks will be associated with lower SSB intake, and this relationship will be stronger after the tax. This would suggest that knowledge is a statistically significant modifier of the change in SSB consumption after the tax in our sample. We expect to see similar effect modification for risk perception. For tax awareness, we expect there to be a statistically significant increase at 12 months given that participants will have noticed increased prices of SSBs in shops. We expect a significantly lower taxed beverage intake among those who are aware of the SSB tax at 12 months compared to those who are unaware. It is possible that there is no difference in awareness at 12 months after the tax compared to baseline, recorded the month before the tax was implemented. This result would suggest that media coverage of the tax has not penetrated into the Langa township, and will provide some explanation for the possibility of finding a small amount of change in Aim 1.

**Limitations**

We are limited in our ability to make causal conclusions from the analyses presented in Aim 2. One of the limitations on causal inference is that we are not able to track individuals over time, so we are restricted to estimating means of two different populations (time 1 versus time 2), rather than to examine changes in the same population of individuals over time. However, there is also an advantage of our cross-sectional study design compared to a longitudinal design for this analysis. Because we have a new set of individuals at subsequent time points, our study design will avoid the bias associated with repeating the same study questionnaire multiple times, which we would expect to be biased towards improved responses over time, after individuals understand the full set of questions to be asked including the negative health implications of high SSB consumption. It is also difficult for us to distinguish whether our results are indicative of true effect modification or whether they are due to social desirability bias. For example, if greater knowledge of the tax increases the likelihood of social desirability bias (i.e. recognizing that drinking SSBs is considered an unhealthy behavior), then those who increase in knowledge may underreport their actual taxed beverage intakes.

**Aim 3: Examine framing of newsprint articles related to the South African SSB tax, whether framing changes over time, and whether framing is associated with proposed causes and solutions for excessive SSB intake.** Beyond their potential impacts on public health, SSB taxes are subject to policy and ethical arguments both in academia^31,92–97^ and in the media^53,62,98^. Media representations of public health policies have a strong effect on public opinion and therefore can affect the likelihood of success or failure of policy proposals^33,55,69^. Media coverage may also increase public awareness of the tax, as we are testing in Aim 2. In this study, we will analyze the response to the SSB tax before and after its implementation in April 2018 to inform SSB policy advocacy in South Africa and across the region. We will perform a quantitative media content analysis of the twenty most widely read English-language online newspapers^99^ across multiple news genres in South Africa for six months preceding and following the SSB tax implementation. We will examine the media response to the tax by coding articles according to the affective attributes (positive, negative, or neutral) and the substantive attributes such as framing of the relationship between SSBs and health outcomes, assignment of responsibility (i.e. individual or industry), and proposed solutions (individual behavior or policy)^100^. We will also examine whether those articles that characterize the problem with excess sugary beverage consumption and weight gain as an individual problem are more likely to propose individualized solutions rather than public health policy solutions.

**Overall Analysis Plan**

The objectives of this aim are (1) to examine the affective attributes (percentage pro/con/neutral) of online newspaper articles writing about the South African SSB tax and whether these change from before to after the tax; (2) examine reasons given for excessive sugar consumption and obesity and whether these causes differ by whether the article is pro, con, or neutral toward the tax; and (3) to test whether pro and con framed articles differ in their proposed solutions to reducing SSB intake (e.g. better education as individual level solution versus taxes as government policy solutions). This analysis will help identify the arguments that are used by either support for or against the SSB tax, which will inform future policymaking.

**Sample Selection**

*Search strategy*

Adapting methods that have been previously described^62,98^, we will conduct a systematic analysis of online news article coverage of the April 2018 SSB tax (called the Health Promotion Levy), using two databases: (1) Nexus Uni, a global database that provides access to full text news, business and legal articles and (2) Google News, a global news aggregator search engine. After our initial searches in these two databases, we will manually search for any potentially missing articles from the top South African newspaper sites listed by Stanford libraries^99^. We will search for articles containing the terms (“tax” OR “levy” OR “health” OR “health promotion”) AND one or more of the following terms: “beverage”, “soft drink”, “soda”, “fizzy drink” AND at least one mention of “sugar”, “sugary”, “sweet”, or “sweetened” AND ("South Africa" OR Johannesburg OR Pretoria OR “Western Cape” OR “Cape Town”). Articles will be included in our search if they were published between October 1, 2017 (6 months before the tax) and October 1, 2019 (6 months after the tax) and were published in English. English is the primary language in South African education, journalism, broadcasting, and advertisements^101^, and news media readership is moderately high in South Africa; 17.5 million (50%) South Africans over the age of 15 read newspapers^102^. We will use the online software product Covidence, commonly used in systematic reviews, to streamline the article review process. Article headlines and abstracts will be read, and clearly irrelevant articles will be excluded.

*Coding*

Our unit of analysis will be the article. We will develop a coding instrument adapted from previous media content analysis work^63^ to assess the presence or absence of supportive (pro) and opposing (con) frames in news coverage about the SSB tax. Pro frames support the policy and argue for either reducing SSB consumption or improving health. Con frames oppose the policy and argue that the SSB tax is regressive (harmful to the poor), harmful to business, or otherwise unfair to certain groups. Articles will be coded as pro, con, or neutral. Neutral articles are either entirely factual or contain both positive and negative frames in equal amounts (>40% of either). To achieve acceptable intercoder reliability, articles will be read by the lead coder and a random 20% subsample will be read and coded by a colleague to ensure intercoder reliability of article content. We will use Krippendorff’s α of 0.8 as a threshold for high agreement^103^.

**Outcomes**

Our key outcomes for this analysis will be the number of pro, con, or neutral articles. We will analyze whether the distribution of these key outcomes differs by time period, proposed cause of high SSB consumption and proposed solution.

**Statistical testing**

First, we will conduct a descriptive analysis of the articles obtained in our search according to whether they are for supporting the tax, against the taxed, or mixed (Table 5). Next, we will conduct proportions testing to test whether there are significant differences in the percentage of positive and negative articles in the time periods before versus after the South African SSB tax.

**Table 5. Framing of news documents covering the South African SSB tax before and after it was passed in April 2018**

| **Source** | **Pro (%)** | **Con (%)** | **Mixed (%)** |
| --- | --- | --- | --- |
| *Pre-tax period total* |  |  |  |
| Source 1 (n = X) |  |  |  |
| Source 2 (n = X) |  |  |  |
| *Post-tax period total* |  |  |  |
| Source 1 (n = X) |  |  |  |
| Source 2 (n = X) |  |  |  |

Next, we will measure the prevalence of different explanations for the cause of excessive SSB consumption and intake of added sugars, following the methods of Buckton and colleagues^62^ (Table 6). We will categorize articles into one of five broad categories: relating to the role of individual decision making for health, the role of industry, the role of society and cultural norms, the role of the government, and the role of scientists (Table 6). Next, we will examine whether these different explanations are associated with support for the tax. We will perform cross-tabulation chi-squared testing to determine whether the percentage of articles supporting the tax differs by the category of cause identified (Table 6).

**Table 6. Frequency of mentioning potential drivers of high SSB consumption and excessive intake from added sugars**

| **Cause** | **n (%)** | **Pro (%)** | **Con (%)** | **Mixed (%)** |
| --- | --- | --- | --- | --- |
| ***Failure of the individual*** |  |  |  |  |
| Individual predisposition (biological, genetic, or psychological) |  |  |  |  |
| Personal choice |  |  |  |  |
| Family background |  |  |  |  |
| Parenting decisions |  |  |  |  |
| Individual dietary habits |  |  |  |  |
| ***Failure of Industry*** |  |  |  |  |
| Inadequate self-regulation of industry and lobbying |  |  |  |  |
| Production and sales of unhealthy products |  |  |  |  |
| Advertising and marketing |  |  |  |  |
| product pricing and pricing promotions |  |  |  |  |
| Insufficient healthy alternatives at restaurants |  |  |  |  |
| ***Failure of society*** |  |  |  |  |
| Geographic areas with few healthy options/abundant fast food |  |  |  |  |
| Entrenched tastes preferences for sweets |  |  |  |  |
| Schools do not provide sufficient nutrition education |  |  |  |  |
| ***Failure of government*** |  |  |  |  |
| Public health education is insufficient |  |  |  |  |
| Unhealthy food environment |  |  |  |  |
| Insufficient regulation or fiscal pressure on industry |  |  |  |  |
| Political behavior |  |  |  |  |
| ***Failure of science*** |  |  |  |  |
| Industry influence on nutrition research |  |  |  |  |
| Problematizing scientific consensus on link between sugar and poor health |  |  |  |  |

Finally, we are interested in whether the pro and con articles differ by the proposed solutions suggested in the articles (Table 7). We will perform cross-tabulation chi-squared testing to determine whether the percentage of articles supporting the tax differs by category of proposed solutions (Table 7).

**Table 7. Analysis plan testing whether there are significant differences between the number of pro and con articles for each proposed solution**

| **Proposed solutions** | **Pro *** | **Con*** |
| --- | --- | --- |
| Individual responsibility | ↓ | ↑ |
| Industry voluntary action | ↑ | ↓ |
| Societal responsibility and changing norms | ↑ | ↓ |
| Government regulation | ↑ | ↓ |
| Role of scientists | ↓ | ↑ |

*Pro and con indicate whether the article is either in favor or against the tax. Arrows indicate predictions for whether pro or con arguments will be more prevalent.

**Expected Results**

We expect that coverage peaked in April 2018 after the passing of the SSB tax, with a significantly greater percentage of anti-tax articles published after the tax as a reaction against it. These negative articles will be more likely to question the role of sugary beverages and added sugars as contributors to weight gain and poor health outcomes. These con articles will be more likely to suggest preferred individual level solutions such as making better choices and exercising more instead of policy solutions, which will be suggested to be economically unjust (e.g. loss of jobs or targeting the poor).

**Limitations**

One of the limitations of this analysis is the ability to make any causal claims about the effects of the news media on how people respond to SSB taxes. Ideally, we would like to have measures of quantity and type of media exposure in our cohort, but this information is not available. As a result, we are only able to describe changes in the overall media landscape in response to the tax and the associations between aspects of media content, not how that content affects individuals because we do not have any measures in our study population that ask about media exposure.

Another limitation is our focus on only English language sources. South Africa has great language diversity, with eleven official languages, many of which are acquired as a first language. It is therefore possible that our results will be biased if coverage of the SSB tax differs between non-English and English sources. The time restrictions on our sampling frame could also limits the number and type of articles that can be considered in this analysis.

**Timeline**

**Table 8. Projected Timeline for Completion of Study Aims**

|  | **2019** | | | | **2020** | |
| --- | --- | --- | --- | --- | --- | --- |
|  | **Q1** | **Q2** | **Q3** | **Q4** | **Q1** | **Q2** |
| **Proposal preparation** |  |  |  |  |  |  |
| **Data Collection and Cleaning** |  |  |  |  |  |  |
| **Aim 1** |  |  |  |  |  |  |
| Analysis using 0, 6, and 12 Month Data |  |  |  |  |  |  |
| **Aim 2** |  |  |  |  |  |  |
| Analysis using 0, 6, and 12 Month Data |  |  |  |  |  |  |
| **Aim 3** |  |  |  |  |  |  |
| Data 6 months prior and following April 2018 |  |  |  |  |  |  |

**References**

1. Popkin, B. M. & Hawkes, C. Sweetening of the global diet, particularly beverages: Patterns, trends, and policy responses. *Lancet Diabetes Endocrinol.* **4,** 174–186 (2016).

2. Malik, V. S., Schulze, M. B. & Hu, F. B. Intake of sugar-sweetened beverages and weight gain: A systematic review. *Am. J. Clin. Nutr.* **84,** 274–288 (2006).

3. Te Morenga, L., Mallard, S. & Mann, J. Dietary sugars and body weight: systematic review and meta-analyses of randomised controlled trials and cohort studies. *Bmj* **346,** e7492–e7492 (2012).

4. Malik, V. S. *et al.* Sugar-Sweetened beverages and risk of metabolic syndrome and type 2 diabetes: A meta-analysis. *Diabetes Care* **33,** 2477–81 (2010).

5. Malik, V. S., Popkin, B. M., Bray, G. A., Despres, J.-P. & Hu, F. B. Sugar-Sweetened Beverages, Obesity, Type 2 Diabetes Mellitus, and Cardiovascular Disease Risk. *Circulation* **121,** 1356–1364 (2010).

6. Arantxa Colchero, M., Rivera-Dommarco, J., Popkin, B. M. & Ng, S. W. In Mexico, evidence of sustained consumer response two years after implementing a sugar-sweetened beverage tax. *Health Aff.* **36,** 564–571 (2017).

7. Caro, J. C. *et al.* Chile’s 2014 sugar-sweetened beverage tax and changes in prices and purchases of sugar-sweetened beverages: An observational study in an urban environment. *PLoS Med.* **15,** 1–19 (2018).

8. Backholer, K., Blake, M. & Vandevijvere, S. Sugar-sweetened beverage taxation: An update on the year that was 2017. *Public Health Nutr.* **20,** 3219–3224 (2017).

9. Park, S., Onufrak, S., Sherry, B. & Blanck, H. M. The relationship between health-related knowledge and sugar-sweetened beverage intake among US adults. *J. Acad. Nutr. Diet.* **114,** 1059–1066 (2014).

10. Kim, B. S., Scheufele, D. A. & Shanahan, J. Think About It This This Way: Attribute Agenda-Setting Function of the Press and the Public’s Evaluation of a Local Issue. *Journal. Mass Commun. Q.* **79,** 7–25 (2002).

11. Kim, S. H. & Willis, L. Talking about Obesity: News framing of who is responsible for causing and fixing the problem. *J. Health Commun.* **12,** 359–376 (2007).

12. National Department of Health. *South African Demographic and Health Survey*. *Statistics South Africa* (2016). doi:10.1378/chest.14-0215

13. SARS. Sugary Beverages Levy. *South African Revenue Service* (2018). at <http://www.sars.gov.za/ClientSegments/Customs-Excise/Excise/Pages/Sugary-Beverages-Levy.aspx>

14. Malik, V. S., Pan, A., Willett, W. C. & Hu, F. B. Sugar-sweetened beverages and weight gain in children and adults: a systematic review and meta-analysis. *Am. Jounral Clin. Nutr.* **98,** 1084–102 (2013).

15. Malik, V. S., Popkin, B. M. & Bray, G. A. Sugar-Sweetened Beverages, Obesity, Type 2 Diabetes Mellitus, and Cardiovascular Disease Risk. *Am. Hear. Assos.* **121,** 1356–1364 (2010).

16. WHO| Guideline Sugars intake for adults and children i Sugars intake for adults and children.

17. Mozaffarian, D. & Al., E. AHA Scientific Statement Population Approaches to Improve Diet, Physical Activity, and Smoking Habits A Scientific Statement From the American Heart Association. *Circulation* **126,** (2012).

18. Bes-Rastrollo, M., Sayon-Orea, C., Ruiz-Canela, M. & Martinez-Gonzalez, M. A. Impact of sugars and sugar taxation on body weight control: A comprehensive literature review. *Obesity* **24,** 1410–1426 (2016).

19. Redondo, M., Hernández-Aguado, I. & Lumbreras, B. The impact of the tax on sweetened beverages: a systematic review. *Am. J. Clin. Nutr.* **108,** 548–563 (2018).

20. Hu, F. Resolved: There is sufficient scientific evidence that decreasing sugar-sweetened beverage consumption will reduce the prevalence of obesity and obesity-related diseases. *Obes. Rev.* **14,** 606–619 (2013).

21. Gallo, A. A Refresher on Price Elasticity. *Harvard Business Review* (2015).

22. Colchero, M. A., Salgado, J. C., Unar-Munguía, M., Hernández-Ávila, M. & Rivera-Dommarco, J. A. Price elasticity of the demand for sugar sweetened beverages and soft drinks in Mexico. *Econ. Hum. Biol.* **19,** 129–137 (2015).

23. Guerrero-López, C. M., Unar-Munguía, M. & Colchero, M. A. Price elasticity of the demand for soft drinks, other sugar-sweetened beverages and energy dense food in Chile. *BMC Public Health* **17,** 1–8 (2017).

24. Sánchez-Romero, L. M. *et al.* Projected Impact of Mexico’s Sugar-Sweetened Beverage Tax Policy on Diabetes and Cardiovascular Disease: A Modeling Study. *PLoS Med.* **13,** 1–17 (2016).

25. Rojas-Martínez, R. *et al.* Expected population weight and diabetes impact of the 1-peso-per-litre tax to sugar sweetened beverages in Mexico. *PLoS One* **13,** e0191383 (2018).

26. Briggs, A. D. M. *et al.* Health impact assessment of the UK soft drinks industry levy: a comparative risk assessment modelling study. *Lancet Public Heal.* **2,** e15–e22 (2017).

27. Veerman, J. L., Sacks, G., Antonopoulos, N. & Martin, J. The impact of a tax on sugar-sweetened beverages on health and health care costs: A modelling study. *PLoS One* **11,** 1–10 (2016).

28. Basu, S. *et al.* Averting Obesity and Type 2 Diabetes in India through Sugar-Sweetened Beverage Taxation: An Economic-Epidemiologic Modeling Study. *PLoS Med.* **11,** (2014).

29. Schwendicke, F. & Stolpe, M. Taxing sugar-sweetened beverages: Impact on overweight and obesity in Germany. *BMC Public Health* **17,** 14–18 (2017).

30. Manyema, M. *et al.* The potential impact of a 20% tax on sugar-sweetened beverages on obesity in South African adults: A mathematical model. *PLoS One* **9,** (2014).

31. Brownell, K. D. *et al.* The Public Health and Economic Benefits of Taxing Sugar-Sweetened Beverages. *N Engl J Med* **361,** 1599–1605 (2011).

32. Basu, S. & Madsen, K. Effectiveness and equity of sugar-sweetened beverage taxation. *PLoS Med.* **14,** 11–14 (2017).

33. Wright, A., Smith, K. E. & Hellowell, M. Policy lessons from health taxes: A systematic review of empirical studies. *BMC Public Health* **17,** 1–14 (2017).

34. Yach, D., Stuckler, D. & Brownell, K. D. Epidemiologic and economic consequences of the global epidemics of obesity and diabetes. *Nat. Med.* **12,** 62–66 (2006).

35. WHO. Obesity and Overweight: Key Facts. *World Health Organization* (2018). at <http://www.who.int/news-room/fact-sheets/detail/obesity-and-overweight>

36. GOV.UK. Guidance: Check if your drink is liable for the Soft Drinks Industry Levy. *HM Revenue & Customs* (2018). at <https://www.gov.uk/guidance/check-if-your-drink-is-liable-for-the-soft-drinks-industry-levy>

37. Bebidas azucaradas, licores, cigarros y productos contaminantes pagarán mayor impuesto. *Agencia Andina* (2018). at <https://andina.pe/agencia/noticia-bebidas-azucaradas-licores-cigarros-y-productos-contaminantes-pagaran-mayor-impuesto-709533.aspx>

38. Jenner, F. Peruvian government puts a 25% tax on sugary drinks to combat rising levels of obesity. *Peru Reports* at <https://perureports.com/peru-sugar-drink-tax/7640/>

39. Lang, T. & Rayner, G. Overcoming policy cacophony on obesity: An ecological public health framework for policymakers. *Obes. Rev.* **8,** 165–181 (2007).

40. Zhong, Y., Auchincloss, A. H., Lee, B. K. & Kanter, G. P. The Short-Term Impacts of the Philadelphia Beverage Tax on Beverage Consumption. *Am. J. Prev. Med.* **55,** 26–34 (2018).

41. Lee, M. M. *et al.* Sugar-Sweetened Beverage Consumption 3 Years After the Berkeley, California, Sugar-Sweetened Beverage Tax. *Am. J. Public Health* e1–e3 (2019). doi:10.2105/AJPH.2019.304971

42. Falbe, J. *et al.* Impact of the Berkeley Excise Tax on Sugar-Sweetened Beverage Consumption. *Am. J. Public Health* **106,** e1–e7 (2016).

43. National Cancer Institute Dietary Assessment Primer: Learn More about Usual Dietary Intake. at <https://dietassessmentprimer.cancer.gov/learn/usual.html>

44. NCI. Dietary Assessment Primer, Evaluating the Effect of an Intervention on Diet. *National Institutes of Health, National Cancer Institute* at <https://dietassessmentprimer.cancer.gov/approach/intervention.html>

45. Silver, L. D. *et al.* Changes in prices, sales, consumer spending, and beverage consumption one year after a tax on sugar-sweetened beverages in Berkeley, California, US: A before-and-after study. *PLoS Med.* 1–19 (2017). doi:10.1371/journal.pmed.1002283

46. Organization, W. H. *Global status report on noncommunicable diseases 2014*. (2014).

47. Cobiac, L. J., Tam, K., Veerman, L. & Blakely, T. Taxes and Subsidies for Improving Diet and Population Health in Australia: A Cost-Effectiveness Modelling Study. *PLoS Med.* **14,** 1–18 (2017).

48. Francis, N., Marron, D. B. & Rueben, K. S. The Pros and Cons of Taxing Sweetened Beverages Based on Sugar Content. *Urban Inst.* (2016). doi:10.2139/ssrn.2947716

49. Veerman, L. The impact of sugared drink taxation and industry response. *Lancet Public Heal.* **2,** e2–e3 (2017).

50. Álvarez-Sánchez, C. *et al.* Does the Mexican sugar-sweetened beverage tax have a signaling effect? ENSANUT 2016. *PLoS One* 1–18 (2018). doi:10.1371/journal.pone.0199337

51. Colchero, M. A., Popkin, B. M., Rivera, J. A. & Ng, S. W. Beverage purchases from stores in Mexico under the excise tax on sugar sweetened beverages: observational study. *BMJ* **352,** h6704 (2016).

52. Donaldson, E. *Advocating for sugar-sweetened beverage taxation: A Case Study of Mexico*. (2015).

53. Buckton, C. H. *et al.* Media representations of sugar and sugar-sweetened beverage consumption in UK newspapers: implications for public health policy. *Lancet* **390,** S27 (2017).

54. Julia, C., Méjean, C., Vicari, F., Péneau, S. & Hercberg, S. Public perception and characteristics related to acceptance of the sugar-sweetened beverage taxation launched in France in 2012. *Public Health Nutr.* **18,** 2679–2688 (2015).

55. Weishaar, H. *et al.* Why media representations of corporations matter for public health policy: A scoping review. *BMC Public Health* **16,** (2016).

56. Henderson, L. & Hilton, S. The media and public health: where next for critical analysis? *Crit. Public Health* **28,** 373–376 (2018).

57. Jacobs, S. & Johnson, K. Media, social movements and the state: Competing images of HIV/AIDS in South Africa. *African Stud. Q.* **9,** 127–152 (2007).

58. Hester, J. B., Gibson, R. & Quarterly, M. C. The economy and second-level agenda setting: A time-series analysis of economic news and public opinion about the economy. *J&MC Q.* **80,** 73–90 (2003).

59. Hilton, S., Patterson, C. & Teyhan, A. Escalating coverage of obesity in UK newspapers: The evolution and framing of the obesity epidemic from 1996 to 2010. *Obesity* **20,** 1688–1695 (2012).

60. Lawrence, R. G. Framing obesity: The evolution of news discourse on a public health issue. *Harvard Int. J. Press.* **9,** 56–75 (2004).

61. Thomas-Meyer, M., Mytton, O. & Adams, J. Public responses to proposals for a tax on sugar-sweetened beverages: A thematic analysis of online reader comments posted on major UK news websites. *PLoS One* **12,** 1–18 (2017).

62. Buckton, C. H. *et al.* The palatability of sugar-sweetened beverage taxation: A content analysis of newspaper coverage of the UK sugar debate. *PLoS One* **13,** e0207576 (2018).

63. Donaldson, E. A. *et al.* News media framing of New York City’s sugar-sweetened beverage portion-size cap. *Am. J. Public Health* **105,** 2202–2209 (2015).

64. Tugendhaft, A. *et al.* Cost of inaction on sugar-sweetened beverage consumption: Implications for obesity in South Africa. *Public Health Nutr.* **19,** 2296–2304 (2016).

65. ALHDC. Langa. *Affordable Land and Housing Data Centre* (2012).

66. Stacey, N., Tugendhaft, A. & Hofman, K. Sugary beverage taxation in South Africa: Household expenditure, demand system elasticities, and policy implications. *Prev. Med. (Baltim).* **105,** S26–S31 (2017).

67. Euromonitor International. at <http://www.euromonitor.com/>

68. Dorfman, L., Wallack, L. & Woodruff, K. More than a message: Framing public health advocacy to change corporate practices. *Heal. Educ. Behav.* **32,** 320–336 (2005).

69. Koon, A. D., Hawkins, B. & Mayhew, S. H. Framing and the health policy process: A scoping review. *Health Policy Plan.* **31,** 801–816 (2016).

70. STATS SA. *Mid-year population estimates 2017*. (2017). doi:Statistical release P0302

71. SAARF. The SAARF AMPS Living Standards Measure (LSM). 92–97 (2011). at <http://www.saarf.co.za/amps-technicalreport/technicalreport-Jan 2011 - Dec 2011/data files/Technical/21 - Tech 2011B ~ Pages 92-97.pdf>

72. SAARF. SAARF Segmentation Tools. *South African Audience Research Foundation2* (2012). at <http://www.saarf.co.za/lsm-presentations/2012/LSM Presentation - February 2012.pdf>

73. Popkin, B. M. *et al.* A new proposed guidance system for beverage consumption in the United States. *Am J Clin Nutr* **83,** 529–42 (2006).

74. Stern, D., Piernas, C., Barquera, S., Rivera, J. A. & Popkin, B. M. Caloric Beverages Were Major Sources of Energy among Children and Adults in Mexico, 1999-2012. *J. Nutr.* **144,** 949–956 (2014).

75. Satija, A., Yu, E., Willett, W. C. & Hu, F. B. Understanding Nutritional Epidemiology and Its Role in Policy. *Adv. Nutr.* 5–18 (2015). doi:10.3945/an.114.007492.5

76. Learn More about Food Composition Databases for 24-hour Dietary Recalls and Food Records. NCI Dietary Assessment Primer. *National Cancer Institute: NIH* at <https://dietassessmentprimer.cancer.gov/learn/recall-record.html>

77. SARS, N. T.-. Final Response Document on the 2017 Rates and Monetary Amounts and Amendment of Revenue Laws Bill - Health Promotion Levy, 2017. (2017). at <http://www.treasury.gov.za/publications/RevenueLaws/2017 Final Response Document-2017 Rates Bill- Health Promotion Levy-15 December 2017.pdf>

78. Willett, W. *Nutritional Epidemiology*. (2013).

79. Masson, L. *et al.* Statistical approaches for assessing the relative validity of a food-frequency questionnaire: use of correlation coefficients and the kappa statistic. *Public Health Nutr.* **6,** 313–321 (2003).

80. Carriquiry, A. Estimation of Usual Intake Distributions of Nutrients and Foods. *J. Nutr.* **133,** 601S–8S (2003).

81. Beaton, G. Sources of variance in 24-hour dietary recall data: implications for nutrition study design and interpretation. *Am. J. Clin. Nutr.* **32,** 2546–2559 (1979).

82. Tooze, J. A. *et al.* A mixed-effects model approach for estimating the distribution of usual intake of nutrients: The NCI method. *Stat. Med.* **29,** 2857–2868 (2010).

83. Tooze, J. A. *et al.* A New Statistical Method for Estimating the Usual Intake of Episodically Consumed Foods with Application to Their Distribution. *J. Am. Diet. Assoc.* **106,** 1575–1587 (2006).

84. Souverein, O. W. *et al.* Comparing four methods to estimate usual intake distributions. *Eur. J. Clin. Nutr.* **65,** S92–S101 (2011).

85. Haubrock, J. *et al.* Estimating Usual Food Intake Distributions by Using the Multiple Source Method in the EPIC-Potsdam Calibration Study. *J. Nutr.* **141,** 914–920 (2011).

86. Harttig, U., Knuppel, S. & Boeing, H. The MSM program: web-based statistics package for estimating usual dietary intake using the Multiple Source Method. *Eur. J. Clin. Nutr.* **65,** S87–S91 (2011).

87. Belotti, F., Deb, P., Manning, W. G. & Norton, E. C. twopm: Two-part models. *Stata J.* **15,** 3–20 (2015).

88. Giannoulis, C. Confirmatory Factor Analysis: How To Measure Something We Cannot Observe or Measure Directly. *The Analysis Factor* (2018). at <https://www.theanalysisfactor.com/confirmatory-factor-analysis-measure-something-we-cannot-observe/>

89. Kenny, D. Measuring Model Fit. (2015). at <http://davidakenny.net/cm/fit.htm>

90. Lewis, T. Fit Statistics commonly reported for CFA and SEM. *Cornell Stat. Dep.* **08,** 0–1 (2017).

91. Maccallum, R. C., Browne, M. W. & Sugawara, H. M. Power Analysis and Determination of Sample Size for Covariance Structure Modeling. *Psychol. Methods* **1,** 130–149 (1996).

92. Buchanan, D. Ethical Standards to Guide the Development of Obesity Policies and Programs. *Int. J. Heal. Policy Manag.* **1,** 313–315 (2013).

93. King, K. F. & Barnhill, A. Fairness and respect in obesity prevention policies: a response to David Buchanan. *Int. J. Heal. policy Manag.* **2,** 49–50 (2014).

94. Barnhill, A., King, K. F., Kass, N. & Faden, R. The Value of Unhealthy Eating and the Ethics of Healthy Eating Policies. *Kennedy Inst. Ethics J.* **24,** 187–217 (2014).

95. Novak, N. L. & Brownell, K. D. Role of policy and government in the obesity epidemic. *Circulation* **126,** 2345–2352 (2012).

96. Brownell, K. D. *et al.* Personal Responsibility And Obesity: A Constructive Approach To A Controversial Issue. *Health Aff.* **29,** 379–387 (2010).

97. Gostin, L. O. Tackling Obesity and Disease: The Culprit Is Sugar; the Response Is Legal Regulation. *Hastings Cent. Rep.* **48,** 5–7 (2018).

98. Elliott-Green, A., Hyseni, L., Lloyd-Williams, F., Bromley, H. & Capewell, S. Sugar-sweetened beverages coverage in the British media: An analysis of public health advocacy versus pro-industry messaging. *BMJ Open* **6,** 1–9 (2016).

99. South Africa news. *Stanford Libraries* at <https://library.stanford.edu/africa-south-sahara/browse-country/south-africa/south-africa-news>

100. Lee, H. & Len-Ríos, M. E. Defining obesity: Second-level agenda setting attributes in black newspapers and general audience newspapers. *J. Health Commun.* **19,** 1116–1129 (2014).

101. Tongues Under Threat. *The Economist* (2011). at <https://www.economist.com/middle-east-and-africa/2011/01/20/tongues-under-threat>

102. AVERAGE ISSUE READERSHIP OF NEWSPAPERS AND MAGAZINES. *South African Audience Research Foundation* at <http://www.saarf.co.za/amps-readership/2008/Readership Summary-08b.pdf>

103. Krippendorff, K. in *The Content Analysis Reader* (eds. Krippendorff, K. & Bock, M.) 350–357 (SAGE Publications, Inc, 2008).
