# Supplementary material for: Taxed and untaxed beverage intake by South African young adults after a national sugar-sweetened beverage tax: A before-and-after study
Source: PLoS Med. 2021 May 25;18(5):e1003574. doi: 10.1371/journal.pmed.1003574 (PMC8148332; doi:10.1371/journal.pmed.1003574)
Supplement: S1 Survey — (PDF) [file pmed.1003574.s003.pdf]

RESEARCHING THE OBESOGENIC FOOD ENVIRONMENT

24 HR RECALL QUESTIONNAIRE

dateint	Date of interview	d d / m m / y y<br>☐☐ / ☐☐ / 1 8	day	Day of the week (yesterday) being recalled	Sun 1 Mon 2 Tues 3 Wed 4 Thurs 5 Fri 6
teamid	Area identifier	Langa 0 3	intidd	Interviewer identifier	☐☐ ……………………
respid	Unique number	yy / mm / dd / country / area / intid / sequence hh<br>___ / ___ / ___ / ___ / ______ / ______	ln	Respondent line number	☐☐
Sex	Sex	M / F	age	Age of respondent	☐☐

ALL INFORMATION WILL BE TREATED CONFIDENTIALY

WHAT FOOD AND DRINKS	HOW WAS IT PREPARED? WHAT WAS ADDED?	HOW MUCH WAS EATEN
**Breakfast time (waking up to about 9 o'clock)**		
**Mid-Morning (09h00 – 12h00)**		
**Lunch Time (12h00 – 14h00)**		

Afternoon (14h00 – 17h00)		
**Supper Time (17h00 – sunset)**		
**After supper, at bedtime and through the night**		

dsp1	Was this a usual day in terms of eating?<br><br>*(CIRCLE ONLY* ***<u>ONE</u>*** *ANSWER.)*	Yes………..................................1<br>No………....................................2	Skip to anthropometry section if answer is yes
dsp2	If not, what was the reason?		

CHECK THE QUESTIONNAIRE & THANK THE RESPONDENT FOR PROVIDING INFORMATION ON YESTERDAY'S FOOD INTAKE. PROCEED WITH SSB CONSUMPTION QUESTIONNAIRE.

**SUPERVISOR INITIAL FOR CHECKING 24HR RECALL**	
**INITIAL FOR CODING OF 24HR RECALL**	

INDIVIDUAL CHARACTERISTICS

respid	Unique number	yy mm dd country area intid sequence hh<br>___ / ___ / ___ / ___ / ______ / ______	ln	Respondent line number	☐☐
Sex	Sex	M / F	age	Age of respondent	☐☐

No.	QUESTIONS	ANSWERS		SKIPS
gc1	How many of the children in this household are your own?		☐☐	
gc2	Do you have any other children who are alive who are not currently living with you in this household?		☐☐	
gc3	What is your employment status?	Unemployed……………………………<br>Self-employed…………………………..<br>Wage earner……………………………<br>Part-time employment…………………<br>Casual worker…………………………...<br>Other (specify)……………………………	1<br>2<br>3<br>4<br>5<br>99	
gc4	What is your marital status?	Single……………………………………..<br>Married …………………………………..<br>Widowed and not remarried……………<br>Divorced and not remarried …………..<br>Married but separated …………………<br>Living together……………………………<br>Other (specify)…………………………..	1<br>2<br>3<br>4<br>5<br>99	Skip to Question he1 if respondent is male
gc5	FOR FEMALE RESPONDENTS<br>How long ago was your last pregnancy?	Never pregnant…………………………..<br>Currently pregnant ………….................<br>Time since last pregnancy in A. years<br>B.months	77<br>0<br>☐☐ or<br>☐☐	Skip to question he1 if time since last pregnancy is >= 2 years/ never
gc6	FOR FEMALE RESPONDENTS<br>Are you currently breastfeeding?	Yes ………………………………………<br>No………………………………………..	1<br>2	Only ask question if time since last pregnancy is <2years

HEALTH

No.	QUESTIONS	ANSWERS		SKIPS
he1	Has a professional ever diagnosed you with one of the following diseases? *(PROMPT FOR EACH ITEM; CIRCLE ONLY ONE ANSWER FOR EACH ITEM)*	Diabetes Mellitus…………………………	Yes…………1<br>No…………..2	
		Heart disease…………………………….	Yes…………1<br>No…………..2	
		High cholesterol……….………………..	Yes…………1<br>No…………..2	
		Hypertension/high blood pressure…….	Yes…………1<br>No…………..2	
		Cancer……………………………………	Yes…………1<br>No…………..2	
		Overweight / Obesity……………………	Yes…………1<br>No…………..2	
he2	Do you currently smoke?	Yes………………………………………<br>No, but smoked previously……………..<br>Yes, occasionally………………………..<br>No, never smoked………………………	1<br>2<br>3<br>4	
he3	What do you smoke most of the time?	Cigarettes……………………………….<br>Pipe………………………………………<br>Hookah pipe……………………………..	1<br>2<br>3	
he4	Do you ever drink any alcohol?	Yes………………………………………<br>No……………………………………….	1<br>2	
he5	Do you ever exercise?	Yes………………………………………<br>No……………………………………….	1<br>2	
he6	How many days a week do you usually do at least 20 minutes the following type of exercise?	A. Walking ……………………………..…<br>B. Moderate exercise…………………<br>C. Vigorous exercise …………………	☐<br>☐<br>☐	
he7	For how many hours in an average week, do you watch movies / television / series/ play electronic games	<1 hour per week…………………………<br>1-3 hrs / week…………………………….<br>4-7hrs / week…………………………….<br>8-14hrs / week (2hrs/day)………………<br>15-21hrs / week (3hrs/day)……………..<br>>21hrs/week………………………………	1<br>2<br>3<br>4<br>5<br>6	
he8	How do you perceive your own weight?	Underweight………………………………<br>Normal weight…………………………….<br>Overweight………………………………..<br>Obese……………………………………..	1<br>2<br>3<br>4	
he9	Is the following statement true or false when you shop for food?	I pay attention to the information on a package like “no sugar added”	True………..1<br>False……….2	

KNOWLEDGE ATTITUDE AND PERCEPTION

(RIvard et al 2012; Madiba et al 2017; Vital Strategies)

I would like to ask you some questions about your understanding of foods and health. Please remember that there is no right or wrong answer, I am just trying to get an understanding of your perception.

No.	QUESTION		ANSWERS	SKIPS
sb1	When you think of the term "sugary drinks" which ones come to mind?<br><br>***(DO NOT PROMPT. PROBE ANYTHING ELSE?)***<br><br>*(CIRCLE YES FOR EACH ITEM MENTIONED AND NO FOR EACH ITEM NOT MENTIONED)*	A. Bottled water (still, sparkling, flavoured)	Yes..............1<br>No…………..2	
		B. 100% fruit juice (eg. Liquifruit, Ceres, Appletiser)	Yes..............1<br>No…………..2	
		C. Nectars or canned juices that contain fruit (eg. Tropicana)	Yes..............1<br>No…………..2	
		D. Milk (full cream, low fat, fat free) (unflavoured)	Yes..............1<br>No…………..2	
		E. Milk (sweetened and flavoured) (eg. Nesquick, Steristumpie, Yogisip)	Yes..............1<br>No…………..2	
		F. Soda or soft drinks (eg. Coca Cola, Sprite, Ginger beer Iron Brew, Dry lemon, Kingsley)	Yes..............1<br>No…………..2	
		G. Diet soda / artificially sweetened beverages (eg. Coca Cola Light, Tab, Diet Sprite)	Yes..............1<br>No…………..2	
		H. Sweetened Iced Tea (eg. BOS, Lipton ice tea, Fuze)	Yes..............1<br>No…………..2	
		I. Coffee/tea with sugar (incl. cappuccino, frapuccino)	Yes..............1<br>No…………..2	
		J. Energy drinks (eg. Red Bull, Monster, Dragon)	Yes..............1<br>No…………..2	
		K. Sports drinks (eg. Energade, Powerade, Lucozade)	Yes..............1<br>No…………..2	
		L. Powdered drinks (eg. Game)	Yes..............1<br>No…………..2	
		M. Cordials and concentrates (eg. Oros)	Yes..............1<br>No…………..2	
		N. Other specify ……………………….	Yes..............1<br>No…………..2	

No.	QUESTION		ANSWERS				SKIPS
sb2	***(PROMPT FOR EACH ITEM)***<br><br>*(MARK ONLY ONE ANSWER FOR EACH ITEM)*		Not sugary (1)	Some what (2)	Sugary (3)	Don't Know (99)	
		A. Bottled water (still)					
		B. Bottled water (sparkling)					
		C. Bottled water (flavoured)					
		D. 100% fruit juice (eg. Liquifruit, Ceres, Appletiser)					
		E. Nectars or canned juices that contain fruit (eg. Tropicana)					
		F. Milk (full cream, low fat, fat free) (unflavoured)					
		G. Milk (sweetened and flavoured) (eg. Nesquick, Steristumpie, Yogisip)					
		H. Soda or soft drinks (eg. Coca Cola, Sprite, Ginger beer, Iron Brew, Dry lemon, Kingsley)					
		I. Diet soda / artificially sweetened beverages (eg. Coca Cola Light, Tab, Diet Sprite)					
		J. Sweetened Iced Tea (eg. BOS, Lipton ice tea, Fuze)					
		K. Coffee/tea with sugar (incl. cappuccino, frapuccino)					
		L. Energy drinks (eg. Red Bull, Monster, Dragon)					
		M. Sports drinks (eg. Energade, Powerade, Lucozade)					
		N. Powdered drinks (eg. Game)					
		O. Cordials and concentrates (eg. Oros)					
		P. Alcohol (beer)					
		Q. Alcohol (wine)					
		R. Alcohol (spirits)					
		S. Alcohol (ciders)					

No.	QUESTION		ANSWERS	SKIPS
sb3	**NOTE: For the purpose of this survey, what we mean by sugary drinks, is all soda or carbonated drinks, energy drinks, sports drinks, flavoured milk or fruit concentrates/nextars or powders for preparing soft drinks. All of them are high in either natural or added sugar**.<br><br>To the best of your knowledge, does the consumption of sugary drinks increase the risk of suffering from …..?<br><br>*(PROMPT FOR EACH DISEASE)*<br><br>*(CIRCLE ONLY ONE ANSWER FOR EACH DISEASE)*	A. HIV	Not at all ….1<br>A little……..2<br>Somewhat..3<br>A lot ………4<br>Not sure/dk.5	
		B. Diabetes	Not at all ….1<br>A little……..2<br>Somewhat..3<br>A lot ………4<br>Not sure/dk.5	
		C. High blood pressure	Not at all ….1<br>A little……..2<br>Somewhat..3<br>A lot ………4<br>Not sure/dk.5	
		D. Obesity	Not at all ….1<br>A little……..2<br>Somewhat..3<br>A lot ………4<br>Not sure/dk.5	
		E. Dental problems	Not at all ….1<br>A little……..2<br>Somewhat..3<br>A lot ………4<br>Not sure/dk.5	
		F. Cancer	Not at all ….1<br>A little……..2<br>Somewhat..3<br>A lot ………4<br>Not sure/dk.5	
sb4	To the best of your knowledge, does obesity increases the risk of suffering from…?	HIV	Not at all……1<br>Just a little …2<br>Somewhat….3<br>A lot…………4<br>Not sure…..99	
		Diabetes (Sugar diabetes)	Not at all……1<br>Just a little …2<br>Somewhat….3<br>A lot…………4<br>Not sure…..99	
		High blood pressure	Not at all……1<br>Just a little …2<br>Somewhat….3<br>A lot…………4<br>Not sure…..99	

	To the best of your knowledge, does obesity increases the risk of suffering from…?		Dental problems		Not at all……1<br>Just a little …2<br>Somewhat….3<br>A lot………..4<br>Not sure…..99	
			Cancer		Not at all……1<br>Just a little …2<br>Somewhat….3<br>A lot………..4<br>Not sure…..99	
sb5	Are you aware of the new Health Promotion Levy (also called Sugary Beverage Tax)?		Yes……………….. 1<br>No………………… 2			
sb6	Do you support the taxation of foods that are less healthy or unhealthy?		Strongly oppose…….. 1<br>Oppose……………… 2<br>Support……………… 3<br>Strongly support……. 4			

			Yes	No	Maybe	Don't know	
sb7	Government has approved a new tax on sugary sweetened beverages which will come into effect on 1 April 2018. If this tax will result in an increase in price of about R2 for 2liters of sugary beverages, how likely will it have the following effect on your purchasing intentions? *(PROMT. MARK ONLY ONE ANSWER FOR EACH OPTION)*	C. I will switch to untaxed drinks					
		B. I will cut back on my sweetened beverage consumption					
		A. I will continue to consume the same drinks. The tax will have no impact at all.					

sb8	If you will cut back or switch to untaxed drinks, which drinks are you most likely to drink more of….? *(DO NOT PROMPT)* *(MARK YES IF AN OPTION IS MENTIONED. MARK NO IF OPTION IS NOT MENTIONED)*	A. Diet soda	Yes…………1<br>No…………..2	
		B. Fruit juice	Yes…………1<br>No…………..2	
		C. Water	Yes…………1<br>No…………..2	
		D. Milk	Yes…………1<br>No…………..2	
		E. Alcohol	Yes…………1<br>No…………..2	
		F. Other (specify) ……………….	Yes…………1<br>No…………..2	

SSB CONSUMPTION AND ACQUISITION

During the past month how often did you (personally) consume any of the following beverages? (*please check the relevant box*) Please read the food category to participants. If they respond positively (i.e. yes we consume this) then ask about the volume most commonly consumed and how often consumed. *Please remember there is no right or wrong answer. We would like to get the most accurate description of the beverages you consumed over the last month. Please tell me about your own consumption of beverages. I would like you to tell me if you drank any of the items that I am going to mention to you. You can also look at the pictures to identify the beverages you drink. If you drink these items, please tell me how often you drink it, and the volume you usually consume.*

**Beverage unit MOST commonly consumed**				
01 200ml	03 440ml	05 750ml	07 1.5l	09 other specify…
02 330ml	04 500ml	06 1l	08 2l	

Code	Food Item	Unit most often consumed	Never <1/mo	1-3/ mo	1/ wk	2-4/ wk	5-6/ wk	1/ day	2-3/ day	≥4/ day
bev1	Water from a tap, not commercial									
bev2	Bottled water (unflavoured) (still, sparkling)									
bev3	Bottled water (flavoured)									
bev4	100% fruit juice (e.g. Liquifruit, Ceres, Appletizer)									
bev5	Nectars or canned juices that contain fruit (e.g. Tropica, Cabana, Halls, Elvin, Dalys, Take5)									
bev6	Cordials/concentrates (e.g. Oros, Fusion, Wild Island)									
bev7	Milk unflavoured and unsweetened (e.g. full cream/low fat/fat free/ amasi)									
bev8	Milk products sweetened & flavored (e.g. Nesquik, Steristumpi, Yogisip)									
bev9	Magheu									
bev10	Regular soda or soft drinks (e.g. Coca Cola, Sprite, Fanta, Stoney Ginger Beer, Cream Soda, Dry Lemon, Jive, Twizza, Refresh)									
bev11	Diet soda/artificially sweetened (e.g. Coca Cola light, Tab, Sprite Zero)									
bev12	Energy drinks (e.g. Score, Red Bull, Monster, Play, Dragon)									
bev13	Sports drinks (e.g. Energade, Powerade, Lucozade)									
bev14	Powdered drinks (e.g. Game)									
bev15	Sweetened iced tea (e.g. BOS Lipton, Fuze)									
bev16	Coffee/tea with sugar (bottled or served; including cappuccino etc)									
bev17	Coffee/Tea without sugar									
bev18	Frozen ice-lollies (e.g. Bompies /JC's)									

Code	Food Item	Unit most often consumed	Never <1/mo	1-3/ mo	1/ wk	2-4/ wk	5-6/ wk	1/ day	2-3/ day	≥4/ day
bev19	Alcohol Wine									
bev20	Alcohol Beer									
bev21	Alcohol Spirits									
bev22	Alcohols Ciders (e.g. Smirnoff Spin)									
bev23	Traditional beer (Umqubothi)									
bev24	Other (specify):									

No.	QUESTION		ANSWER	
bev25	Have you changed your beverage consumption because of the drought in the Western Cape?		Yes…………1<br>No…………..2	
bev26	If yes, please tell me what you drink more or less of as a result of the drought….	A. Drink more of…(specify)		
		B. Drink less of ….(specify)		

THANK THE PARTICIPANTS FOR THEIR TIME
SHARE TOKEN OF APPRECIATION WITH THE HOUSEHOLD
