# Supplementary material for: Taxed and untaxed beverage intake by South African young adults after a national sugar-sweetened beverage tax: A before-and-after study
Source: PLoS Med. 2021 May 25;18(5):e1003574. doi: 10.1371/journal.pmed.1003574 (PMC8148332; doi:10.1371/journal.pmed.1003574)
Supplement: S1 Table — (DOCX) [file pmed.1003574.s004.docx]

**S1 Table. Beverage classification system**

| **Level 1** | **Level 2** | **Level 3** |
| --- | --- | --- |
| All beverages | Carbonates | < 4 g/100ml Untaxed |
|  |  | ≥ 4 g/100ml Taxed |
|  | Fruit Drinks & Nectars | < 4 g/100ml Untaxed |
|  |  | ≥ 4 g/100ml Taxed |
|  | Concentrates | < 4 g/100ml Untaxed |
|  |  | ≥ 4 g/100ml Taxed |
|  | Sports & Energy Drinks | < 4 g/100ml Untaxed |
|  |  | ≥ 4 g/100ml Taxed |
|  | Flavored Waters | < 4 g/100ml Untaxed |
|  |  | ≥ 4 g/100ml Taxed |
|  | Bottled and Flavored Waters | < 4 g/100ml Untaxed |
|  |  | ≥ 4 g/100ml Taxed |
|  | Milks (sweetened) | < 4 g/100ml Untaxed |
|  |  | ≥ 4 g/100ml Taxed |
|  | Coffee/Tea | All untaxed, sugar added at home |
|  | Milks (unsweetened) | Exempt, all untaxed |
|  |  |  |
|  | 100% Fruit Juice | Exempt, all untaxed |
|  |  |  |
|  | Plain waters | All untaxed |
