# Supplementary material for: Taxed and untaxed beverage intake by South African young adults after a national sugar-sweetened beverage tax: A before-and-after study
Source: PLoS Med. 2021 May 25;18(5):e1003574. doi: 10.1371/journal.pmed.1003574 (PMC8148332; doi:10.1371/journal.pmed.1003574)
Supplement: S2 Table — (DOCX) [file pmed.1003574.s005.docx]

**S2 Table. Model adjusted predicted intake of total sugar for taxed and untaxed beverage subcategories, Langa adults 18-39y**

|  | Pre-tax | Post-tax |
| --- | --- | --- |
| Beverage category | Total Sugar grams per capita | Total Sugar grams per capita |
|  | Mean (95% CI) | Mean (95% CI) |
| Taxed | 28.8 (27.3 to 30.4) | 19.8 (18.5 to 21.1) |
| Flavored waters | <0.5 | <0.5 |
| Carbonates | 19.6 (18.2 to 21.0) | 17.2 (15.9 to 18.4) |
| Fruit Drinks & Nectars | 7.3 (6.5 to 8.1) | 1.5 (1.1 to 1.8) |
| Concentrates | 0.8 (0 to 1.6) | 0.0 (0 to 0.1) |
| Sports & Energy | 1.1 (0.8 to 1.5) | 0.9 (0.6 to 1.2) |
| Dairy and dairy substitutes (flavored, sweetened) | 0.4 (0.0 to 2.8) | 0.5 (0 to 5.4) |
| Untaxed | 15.0 (13.9 to 16.0) | 20.3 (19.2 to 21.4) |
| Plain water | 0 | 0 |
| Flavored low sugar waters (<4g/100ml) | <1 | <1 |
| Dairy and dairy substitutes (unflavored, unsweetened) | 2.9 (2.7 to 3.2) | 4.2 (3.9 to 4.6) |
| 100% fruit juice | 0.5 (0.2 to 0.9) | 0.5 (0.1 to 0.9) |
| Carbonates | 0 | 1.1 (0.8 to 1.5) |
| Fruit Drinks & Nectars | 0 | 0.1 (0.0 to 0.2) |
| Concentrates | 0.7 (0.5 to 0.8) | 1.5 (1.3 to 1.8) |
| Coffee/Tea (all sugar content) | 10.8 (9.8 to 11.8) | 12.5 (11.4 to 13.6) |
| Sports & Energy | <1 | <1 |
| Total beverages | 43.8 (41.9 to 45.7) | 40.1 (38.5 to 41.6) |

From models adjusting for age, sex, weekday versus weekend, and average daily temperature. Values in parentheses represent 95% Confidence Intervals (CI).
