# Supplementary material for: Taxed and untaxed beverage intake by South African young adults after a national sugar-sweetened beverage tax: A before-and-after study
Source: PLoS Med. 2021 May 25;18(5):e1003574. doi: 10.1371/journal.pmed.1003574 (PMC8148332; doi:10.1371/journal.pmed.1003574)
Supplement: S3 Table — (DOCX) [file pmed.1003574.s006.docx]

**S3 Table. Model adjusted predicted intake of energy (kcal) for taxed and untaxed beverage subcategories, Langa adults 18-39y**

|  | Pre-tax | Post-tax |
| --- | --- | --- |
| Beverage category | kcal per capita | kcal per capita |
|  | Mean (95% CI) | Mean (95% CI) |
| Taxed | 121 (114 to 127) | 82 (76 to 87) |
| Flavored waters | <1 | <1 |
| Carbonates | 79 (74 to 85) | 69 (64 to 74) |
| Fruit Drinks & Nectars | 32 (29 to 36) | 7 (5 to 8) |
| Concentrates | 3 (0 to 7) | 0 (0 to 1) |
| Sports & Energy | 5 (4 to 7) | 4 (3 to 5) |
| Dairy and dairy substitutes (flavored, sweetened) | 2 (0 to 9) | 1 (0 to 6) |
| Untaxed | 105 (99 to 112) | 135 (128 to 141) |
| Plain water | 0 | 0 |
| Flavored low sugar waters (<4g/100ml) | <1 | <1 |
| Dairy and dairy substitutes (unflavored, unsweetened) | 49 (45 to 54) | 62 (57 to 67) |
| 100% fruit juice | 2 (1 to 4) | 2 (1 to 4) |
| Carbonates | 0 | 5 (3 to 6) |
| Fruit Drinks & Nectars | 0 | <1 |
| Concentrates | 3 (2 to 4) | 6 (5 to 7) |
| Coffee/Tea (all sugar content) | 51 (46 to 55) | 57 (52 to 62) |
| Sports & Energy | <1 | <1 |
| Total beverages | 226 (217 to 235) | 216 (208 to 224) |

From models adjusting for age, sex, weekday versus weekend, and average daily temperature. Values in parentheses represent 95% Confidence Intervals (CI).
