# Supplementary material for: Taxed and untaxed beverage intake by South African young adults after a national sugar-sweetened beverage tax: A before-and-after study
Source: PLoS Med. 2021 May 25;18(5):e1003574. doi: 10.1371/journal.pmed.1003574 (PMC8148332; doi:10.1371/journal.pmed.1003574)
Supplement: S4 Table — (DOCX) [file pmed.1003574.s007.docx]

**S4 Table. Model adjusted predicted intake of volume for taxed and untaxed beverage subcategories, Langa adults 18-39y**

|  | Pre-tax | Post-tax |
| --- | --- | --- |
| Beverage category | mL per capita | mL per capita |
|  | Mean (95% CI) | Mean (95% CI) |
| Taxed | 315 (297 to 332) | 198 (185 to 211) |
| Flavored waters | 1 (0 to 2) | 2 (0 to 3) |
| Carbonates | 212 (197 to 227) | 171 (159 to 183) |
| Fruit Drinks & Nectars | 80 (71 to 90) | 14 (11 to 18) |
| Concentrates | 8 (0 to 16) | 0 (0 to 1) |
| Sports & Energy | 12 (8 to 16) | 9 (6 to 12) |
| Dairy and dairy substitutes (flavored, sweetened) | 1 (0 to 1) | <1 (0 to 1) |
| Untaxed | 587 (563 to 610) | 926 (899 to 953) |
| Plain water | 342 (321 to 363) | 519 (498 to 540) |
| Flavored low sugar waters (<4g/100ml) | <1 | <1 |
| Dairy and dairy substitutes (unflavored, unsweetened) | 74 (67 to 81) | 101 (93 to 109) |
| 100% fruit juice | 5 (2 to 7) | 5 (2 to 7) |
| Carbonates | 1 (0 to 2) | 36 (25 to 47) |
| Fruit Drinks & Nectars | 0 | 7 (0 to 15) |
| Concentrates | 28 (22 to 33) | 116 (106 to 125) |
| Coffee/Tea (all sugar contents) | 138 (127 to 148) | 137 (127 to 147) |
| Sports & Energy | <1 | <1 |
| Total beverages | 901 (876 to 927) | 1124 (1097 to 1151) |

From models adjusting for age, sex, weekday versus weekend, and average daily temperature. Values in parentheses represent 95% Confidence Intervals (CI).
