# Supplementary material for: Taxed and untaxed beverage intake by South African young adults after a national sugar-sweetened beverage tax: A before-and-after study
Source: PLoS Med. 2021 May 25;18(5):e1003574. doi: 10.1371/journal.pmed.1003574 (PMC8148332; doi:10.1371/journal.pmed.1003574)
Supplement: S5 Table — (DOCX) [file pmed.1003574.s008.docx]

**S5 Table. Model adjusted predicted intakes of total sugar, energy, and volume from taxed beverages for each LSM category**

| **LSM Category** | **Total Sugar  g/capita/day**  **(95% CI)** | | **Energy  kcal/capita/day**  **(95% CI)** | | **Volume  mL/capita/day**  **(95% CI)** | |
| --- | --- | --- | --- | --- | --- | --- |
| **Taxed**  **Beverages** | Pre-tax | Post-tax | Pre-tax | Post-tax | Pre-tax | Post-tax |
| 3 and 4 | 28.6  (24.9 to 32.2) | 18.6**  (15.7 to 21.5) | 121 (106 to 137) | 76** (64 to 88) | 311 (271 to 350) | 184** (156 to 213) |
| 5 | 29.5  (27.1 to 31.9) | 20.4**  (18.5 to 22.3) | 123  (113 to 133) | 84**  (77 to 92) | 322  (296 to 348) | 204**  (185 to 222) |
| 6 | 28.3  (26.0 to 30.6) | 19.7**  (17.3 to 22.1) | 119  (109 to 128) | 81**  (71 to 91) | 309  (283 to 334) | 199**  (175 to 223) |
| **Untaxed**  **Beverages** |  |  |  |  |  |  |
| 3 and 4 | 13.8  (11.5 to 16.1) | 19.3*  (16.9 to 21.7)  p=0.004 | 102  (87 to 118) | 127*  (113 to 141)  p=0.005 | 588  (532 to 644) | 968**  (908 to 1028) |
| 5 | 14.1  (12.6 to 15.7) | 20.6**  (19.1 to 22.1) | 100  (90 to 110) | 134**  (126 to 143) | 586  (550 to 622) | 890**  (854 to 925) |
| 6 | 16.6  (14.9 to 18.3) | 20.3**  (18.2 to 22.3) | 114  (103 to 124) | 138**  (126 to 150) | 590  (556 to 623) | 954**  (903 to 1005) |
| **Total**  **Beverages** |  |  |  |  |  |  |
| 3 and 4 | 42.2  (37.8 to 46.6) | 37.9  (34.7 to 41.2) | 223  (201 to 245) | 203  (186 to 220) | 901  (840 to 962) | 1152*  (1093 to 1212)  p=0.007 |
| 5 | 43.4  (40.6 to 46.3) | 41.1  (38.9 to 43.2) | 223  (209 to 237) | 219  (208 to 230) | 909  (870 to 948) | 1093*  (1056 to 1129)  p=0.003 |
| 6 | 45.0  (42.1 to 47.8) | 39.9  (37.1 to 42.7) | 232  (218 to 246) | 219  (204 to 233) | 898  (861 to 935) | 1154*  (1103 to 1206)  p=0.001 |

From models adjusting for age, sex, weekday versus weekend, and average daily temperature. Values in parentheses represent 95% Confidence Intervals (CI). P-values relate to within-row comparisons (post-tax compared to pre-tax). Exact p-values are reported unless p<0.001.

*Indicates statistical significance within row (post-tax compared to pre-tax) at p<0.01 level

**Indicates statistical significance within row (post-tax compared to pre-tax) at p<0.0001 level
