# Supplementary material for: Taxed and untaxed beverage intake by South African young adults after a national sugar-sweetened beverage tax: A before-and-after study
Source: PLoS Med. 2021 May 25;18(5):e1003574. doi: 10.1371/journal.pmed.1003574 (PMC8148332; doi:10.1371/journal.pmed.1003574)
Supplement: Attachment — Submitted filename: PMEDICINE-D-20-03807R1 responses to reviewers final.docx [file pmed.1003574.s009.docx]

**PMEDICINE-D-20-03807R1**

**Requests from the editors:
Please revise your title according to PLOS Medicine's style. Your title must be nondeclarative and not a question. It should begin with main concept if possible. "Effect of" should be used only if causality can be inferred, i.e., for an RCT. Please place the study design ("A randomized controlled trial," "A retrospective study," "A modelling study," etc.) in the subtitle (i.e., after a colon).**

Our response: We have revised the title to “Changes in taxed and untaxed beverage intake by South African young adults after a national sugar-sweetened beverage tax: a before-and-after study”

**Abstract
Could we perhaps rephrase “growing burdens of obesity and diabetes” I wonder if this is a bit stigmatising and could be revised**

Our response: Thank you for this comment. We have changed the phrase to “In an effort to prevent and reduce the prevalence rate of people with obesity and diabetes” to remove the stigmatizing language and make the sentence more descriptive.

**Please clarify what you mean here “We estimated beverage intake using a two-part model, with a probit model for the first part and a generalized linear model with log-link for the second part”. Please also outline briefly how you planned to test behavioural versus reformulation effects**

Our response (two-part model): We have clarified how the two-part model works by explaining each step. We changed the language to “We estimated beverage intake using a model that takes into consideration the biases in using an OLS or other continuous variable approach with many individuals with zero intake, namely a two-part model. First, a probit model was used to estimate the probability to consume the specific beverage category. Then, conditional on a positive outcome, a generalized linear model with a log-link was used to estimate the continuous amount of beverage consumed.”

Our response (testing behavioural versus reformulation effects): Thank you for this comment, we have added to the explanation of the food composition tables used to separate behavioural change from reformulation: “We developed time specific food composition tables (FCTs) for South African beverages before and after the tax, linked with the diet recalls. By linking pre-tax FCTs only to dietary intake data collected at pre-tax and post-tax periods, we calculated changes in beverage intake due to behavioral change, assuming no reformulation. Next, we repeated the analysis using an updated FCT in the post-tax period to capture the marginal effect of reformulation.”

**The Data Availability Statement (DAS) requires revision. For each data source used in your study:

a) If the data are freely or publicly available, note this and state the location of the data: within the paper, in Supporting Information files, or in a public repository (include the DOI or accession number).

b) If the data are owned by a third party but freely available upon request, please note this and state the owner of the data set and contact information for data requests (web or email address). Note that a study author cannot be the contact person for the data.

c) If the data are not freely available, please describe briefly the ethical, legal, or contractual restriction that prevents you from sharing it. Please also include an appropriate contact (web or email address) for inquiries (again, this cannot be a study author).**

Our response: The original South African food composition table is owned by the South African Medical Research Council. They are the only group that can disseminate the data. We added to this detailed data in both the pre and post period FCT’s for all the specific SSB’s and other beverages consumed in South Africa through data collected from supermarkets. For the baseline we worked with a set of data collected by the South African insurance company Discovery and its prevention arm, the Vitality Program in collaboration with the George Institute. We used their software to collect the data. We cannot share under an agreement with Vitality.

For the follow-up we worked with a system developed by the Global Food Research Program at the University of North Carolina. We cannot make these data publicly available but can share if users sign a confidentiality agreement and are not involved with the food industry.  **At this stage, we ask that you include a short, non-technical Author Summary of your research to make findings accessible to a wide audience that includes both scientists and non-scientists. The Author Summary should immediately follow the Abstract in your revised manuscript. This text is subject to editorial change and should be distinct from the scientific abstract. Please see our author guidelines for more information:** [**https://journals.plos.org/plosmedicine/s/revising-your-manuscript#loc-author-summary**](https://journals.plos.org/plosmedicine/s/revising-your-manuscript#loc-author-summary)

Our response:
Why Was This Study Done?

- In 2018, South Africa became the first sub-Saharan African country to implement a sugary beverage tax to reduce consumption of added sugars.
- SSB taxes have been shown to reduce purchases, but few studies have included dietary intake, and they are focused in higher-income countries like the United States. This is the first study to examine the effects of an SSB tax using dietary intake data focused on a low-income population from the Global South.
- This is the first study to evaluate real world changes from an SSB tax policy by empirically quantifying how much of the overall change in sugar from taxed beverages came from consumers’ behavioral changes versus reformulation of beverages.

What Did the Researchers Do and Find?

- Using dietary intake data collected from a low-income South African township before and after the SSB tax was implemented, we examined changes in sugar, calories and volume from taxed and untaxed beverages.
- We used detailed brand-product-specific nutrition facts panel data collected at baseline and a year after tax implementation to allow us to examine both behavioral change and reformulation.
- We found the intake of sugar, calories and volume of taxed beverages decreased by 9.0 g (31%), 39 kcal (33%), and 117 mL (37%) per capita per day, respectively. The intake of sugar, calories and volume of taxed beverages increased by 5.3 g (36%), 30 kcal (29%), and 339 mL (58%) per capita per day, respectively. The majority of this increase (52%) was due to increased water consumption.
- Of the 9.0 g per capita per day (31%) total reduction in sugar from taxed beverages, 6.4 g per capita per day (22%) was due to behavioral change, and an additional 2.6 g per capita per day (9%) reduction was due to reformulation.

What Do These Findings Mean?

- SSB taxes were associated with reduced sugary drink intake in a low-income population within a middle-income country.
- By examining the two primary goals of an SSB tax based on sugar concentration, we quantified the observed changes in beverage intake due to reformulation and behavior change.
- Future research will be needed to understand how responses to sugar-based beverage taxes may vary by socioeconomic status.

**Please use square brackets for references**
Our response: Thank you for this comment. We have changed references to square brackets.  **Please add p-values of up to three decimal places, providing exact values throughout unless p<0.001**
Our response: Thank you for this comment. We have changed reported p values to consistently follow these guidelines, reporting exact p value to three decimal places unless p<0.001 **Please provide copies of questionnaires used or citations if they have been previously published**

Our response: We have uploaded the questionnaire along with the other submission materials.

**Did your study have a prospective protocol or analysis plan? Please state this (either way) early in the Methods section.

a) If a prospective analysis plan (from your funding proposal, IRB or other ethics committee submission, study protocol, or other planning document written before analyzing the data) was used in designing the study, please include the relevant prospectively written document with your revised manuscript as a Supporting Information file to be published alongside your study, and cite it in the Methods section. A legend for this file should be included at the end of your manuscript.

b) If no such document exists, please make sure that the Methods section transparently describes when analyses were planned, and when/why any data-driven changes to analyses took place.

c) In either case, changes in the analysis-- including those made in response to peer review comments-- should be identified as such in the Methods section of the paper, with rationale.**

Our response: The first author’s approved dissertation proposal laid out the detailed analysis plan followed for this paper. This was also approved in broader terms by the IRB of UNC. We have also added to the beginning of the Methods section, “This study was part of a student dissertation and not registered in a public repository. However, the original proposal of the study design and analytical plan from the dissertation proposal can be found in Appendix A.” The dissertation proposal consists of 3 projects, of which this submitted manuscript is one. Aim 1 is the only relevant section to this manuscript under consideration, which focuses on examining changes in beverage intake after the HPL. This project does not include the examination of modifiers, which is the goal of a different project. The methods relevant to this project begin on page 11 with Aim 1, and end on page 14. We have included the entire dissertation proposal for full transparency as it was submitted.

For the manuscript, we added the following to the start of the Analytical Approach section of the Methods, “There were few deviations from the initial Analysis Plan outlined in Appendix A. For this study, we added total beverages as an outcome to demonstrate the total effect of the policy on beverages, not just taxed and untaxed beverages alone. We do not report results from beverage subcategories to maintain focus and clarity on changes in taxed, untaxed, and total beverage groups after the HPL.**”

Table 1- I suggest you add the units for “Age”**
Our response: Thank you for this comment. We have modified the table to Age (years).
 **Discussion- please start this section with a brief description of what was done and the main findings**

Our response: Thank you for this comment. We added more description of the study conducted in the first paragraph of the Discussion and also included more findings for untaxed beverages.  **I suggest the discussion is tempered, by adding “our results indicate” rather than “we show” due to the observational nature of this study**
Our response: Thank you for this comment. We have changed “we show” to “our results indicate,” and later in the Discussion (paragraph 4) “this study found” was changed to “our results indicate.” **Please also temper all assertions of primacy by adding “to our knowledge”**
Our response: Thank you for this comment. We have qualified these assertions by starting with “to our knowledge” including paragraphs 2 and 3 of the Discussion.
 **Please remove the funding information from the main text and add this information to the financial disclosure section to the article meta-data**
Our response: We have removed the funding information from the main text and included it in the financial disclosure section of the article meta-data.  **Please ensure that the study is reported according to the STROBE guideline, and include the completed STROBE checklist as Supporting Information. Please add the following statement, or similar, to the Methods: "This study is reported as per the Strengthening the Reporting of Observational Studies in Epidemiology (STROBE) guideline (S1 Checklist)."

The STROBE guideline can be found here:** [**http://www.equator-network.org/reporting-guidelines/strobe/**](http://www.equator-network.org/reporting-guidelines/strobe/) **When completing the checklist, please use section and paragraph numbers, rather than page numbers.**

Our response: We have completed and uploaded the STROBE checklist with the submission materials.  **Comments from the reviewers:

Reviewer #1: This is an excellent manuscript that provides a thorough evaluation of an important population health intervention. The methods that are reported are novel, and the focus on the low income group is important. This paper makes an excellent contribution to the literature.

I have some minor comments:
1.      If the pre-tax data were collected two months before the implementation of the tax then some reformulation of soft drinks could have taken place already, which would result in an underestimate of the impact of the tax on sugar consumption. Our analysis of the impact of the UK sugar drink levy on sugar levels in drinks suggested that two months before the implementation of the levy the proportion of drinks with sugar levels over the 5g per 100mL levy threshold had already fallen by about 20 percentage points (Scarborough et al. PLoS Med, 2020,** [**https://doi.org/10.1371/journal.pmed.1003025**](https://doi.org/10.1371/journal.pmed.1003025)**).**

Our response: Thank you for this comment on the interpretation of our results. In light of comments from another reviewer, we have added more emphasis in the Discussion on the total effect of the tax on beverage intake—i.e. accounting for both taxed reductions and untaxed increases. However, we have also noted the effects found in this study could be an underestimation of the reformulation effect in paragraph 4 of the discussion, citing the Scarborough et al. PLoS Med 2020 paper^1^ and also the Bandy et al paper from BMC Medicine.^2^ It was unfortunately not possible to design the study and collect the dietary intake and nutrition facts information quickly enough to capture changes in the beverage supply between the announcement and the SSB tax implementation. It was more feasible to do this in our Kantar food purchase analysis [separately under review], which accounted for the pre-implementation post tax announcement period like the two Scarborough et al papers.^1,2^ We have also added this detail to paragraph 4 of the discussion: “Our study did not capture any pre-emptive reductions in sugar content that have been suggested in other countries. Other data sources such as purchase data will be important for capturing changes in nutrition content of beverage in the time period between announcement and implementation of the tax.”
 **2.      Lines 15—167: The description of how food diary data are linked with the nutrient composition tables is not very clear, and would be helped by an example. From my understanding, in the food diary data there was sometimes ambiguity at a brand-level (e.g. "Coca-Cola" could cover regular, cherry, mango, vanilla, etc.), and these multiple options were dealt with by weighting using Kantar World Panel data on consumption. If that is the case, then it would be useful to know whether this ambiguity extended to inclusion of both diet (i.e. zero calorie variants) and regular drinks amongst the same brand, which would be a big limitation.**

Our response: Thank you for this comment. You are correct in summarizing the need for weighting by Kantar World Panel if there was ambiguity by brand, but we were very careful to always collect diet vs regular beverages in our data collection for each type of beverage, as this would affect whether the beverage was placed in the beverage code that belonged above or below 4g/100mL taxation threshold. We added this detail to paragraph 5 of the Methods, under *Linking Food Composition Tables to Beverage Categories*. **3.      The percentage split of men and women at each timepoint in table 1 are identical. Just checking whether this is a typo!**

Our response: Thank you for this comment and for double checking. They were very close. They are identical to the tenths decimal place due to rounding.

Pre-tax period

Post-tax period

**4.      Lines 282-290: This should start "For total beverages" rather than "For untaxed beverages". However, the next bit of text does refer to 'untaxed beverages, so this paragraph needs a little bit of editing.**

Our response: Thank you for this comment and noticing this editing error. We fixed the portion relevant to total beverages and moved it to after the untaxed portion (now begins line 377) to fit the same logical flow we used for other results: taxed, untaxed, then total beverages.  **5.      It is not clear to me how reformulation can have affected the volume of drink consumed. The only mechanism that I can think is that the respondents recorded how many units of drink they consumed (e.g. a bottle, a can) and the size of these units changed as a result of the tax. If so, then that should be made clear in the methods. Also, how were these units linked to specific serving sizes (e.g. in the UK, Coca-Cola is available in 250mL and 330mL cans. If a respondent reports consumption of 'a can of Coca-Cola' how do you choose which serving size to assign?)**

Our response: Thank you for this comment. Given that our decomposition method is based on inference—that is, the total effect of the policy is the sum of the effects of behavioral change plus reformulation—the interpretation of volume can be confusing. In the first case, for behavioral change alone, we estimate an effect assuming none of the beverage categories either reduced their sugar content or changed taxation categories due to reformulation. However, using the updated FCT, we are allowing for changes in consumer behavior, changes in sugar content within beverage categories, as well as changing of beverage across taxation categories. Therefore, if a squash beverage were taxed in the pre- period but theoretically consumed at the same level, there would be no change according to behavior. However, if some squash products reported were later classified in the untaxed category due to reformulation, this would be apparent using the updated FCT. Therefore, given the same state of the world in terms of what people consumed, accounting for reformulation could then shift the volume captured by the taxed and untaxed categories. We have added this explanation to paragraph 3 of the Discussion: “Changes in volume due to reformulation should be interpreted as effects of updating sugar concentration that led to changing taxed beverage classification. For example, a beverage from the pre-tax period could become categorized as untaxed if it was reformulated below the 4g/100mL threshold.”

For the latter portion of the question about serving size with the example of 250mL and 330mL cans given, fieldworkers who conducted the dietary recalls were trained to prompt for volume size, and had in their field kit a collection of standardized cup sizes to help respondents best approximate the volume of beverage consumed. All beverages consumed were therefore reported with a volume in mL.  **6.      I would like more information about how the nutrition data were collected. The manuscript simply says "First, nutrition facts panel (NFP) data were collected from South African grocery stores in February and March 2018. This was repeated exactly a year later in February and March 2019." How were the stores sampled? How were the products sampled? Was a complete audit conducted, or were only drinks that appeared in the food diary included?**

Our response: Thank you for this comment. To the same paragraph, the fifth of the Methods section, we added, “The stores were sampled in two ways. First, we obtained permission and collected all NFP data and other information from beverages at all major chains functioning in the Cape Town region. Second, we visited small stores in the sample township area to ensure we collected any beverages missed in the chain stores that may have been consumed by our sample population.” **Reviewer: Peter Scarborough


Reviewer #2: Thank you for the opportunity to review "Taxed and untaxed beverage consumption by young adults in Langa, South Africa before and one year after a national sugar-sweetened beverage tax." This manuscript uses a before and after study design to estimate the effect of the South African SSB tax on the consumption of sugar, energy and volume of both taxed and untaxed beverages, in a low-income adult community. The main strength of the paper is the use of detailed 24-hour dietary recalls linked with updated food composition databases for beverage. The paper is very clear, well structured and well written.
In my read, the main limitation of the paper is the pre/post study design, as differences observed could be due to all sorts of time-varying factors, such as differences in the population sampled (either due to sampling process, change within the population, or change of the population, such as rising income), pre-existing decreasing trends in SSB purchases, changes in accessibility of beverages. In addition, the authors mention that there was a drought and severe water use restriction during part of the baseline data collection period. To account for some of these confounding effects, the authors adjust their results for a limited number of variables available, incl. age, day of the week, temperature, and a measure of socio-economic status, yet this does not rule out the presence of residual bias, such that the strength of the policy effect may remain uncertain.

My specific comments and suggestions are as follows
1.      Results summary in the abstract and conclusion: in my read, there seems to be a mismatch between what the paper says it intends to do and the results highlighted in the abstract. Both the introduction and the abstract indicate that the study aims to estimate changes in taxed and untaxed beverage intake. However, the only results presented in the abstract highlight the decrease in taxed beverage intake, showing that the tax might have had a positive effect on sugar and energy reduction. However, more than half of the reduction observed is compensated by what appears to be substitution towards untaxed beverages, which have dramatically increased in terms of overall volume consumption. This means that all in all, the SSB tax resulted in a relatively small reduction in sugar intake and an uncertain reduction in total energy from beverages in that population.**

Our response: Thank you for this comment. We have revised the abstract to present all of the results including taxed, untaxed, and total beverages. In the conclusion, we added, “This reduction was partially compensated by an increase in sugar and energy from untaxed beverages.” **2.      I would remove the emphasis on the fact that looking at differences between reformulation and behavioural change is a novelty of this paper. Rachel Griffith et al. proposed a more sophisticated decomposition method in a 2014 IFS working paper "The importance of product reformulation versus consumer choice in improving diet quality". The paper makes the distinction between changes due to reformulation, behavioural change and introduction of new products. The method was applied to beverages by Marine Spiteri & Louis-Georges Soler in paper published in the European Journal of Clinical Nutrition in 2017 ("Food reformulation and nutritional quality of food consumption: an analysis based on households panel data in France").** 

Our response: Thank you for this comment. The paper by Griffith and colleagues used a decomposition method that was useful for measuring changes in salt density of food purchases. This method and study goal were useful for determining relative changes in the food supply, but their stated goal was not to examine the effect of a real world policy on total changes in salt ***intake***. Economists commonly refer to consumption as inclusive of purchases and intake, but our study was solely focused on dietary intake. Thus, we find the interpretability of the Griffith paper different from ours as it has a different set of study goals.

To measure the effects of reformulation, the Griffith and Spiteri papers focus on disaggregating three components: reformulation of existing food products by manufacturers, the net effect of the launching/removal of products, and consumer switching between products. Because our study is focused on the changes in total beverage intake after the policy, we only attempt to disaggregate the effects of consumer behavior change from the total effects of reformulation. Quantifying relative changes in average nutrient content for newly introduced, existing, and discontinued products was not necessary to achieve our study goal. Using our approach consisting of dietary intake data with a focus on total intake, we are not applying a mathematical approach, rather using inference based on findings. I should add that getting a real world interpretation from this excellent Spiteri study is not straightforward given their focus on nutrient density, not total nutrient intake. Our study, in contrast, provides a very simple translation of the results in grams of sugar or kcal per capita per day.

We find that our differences in research questions means our approach is still appropriate for our goals. This is the first paper both in the African context and for evaluating a novel SSB tax design based on sugar-context to use our method. However, our method does have limitations. The nutrient content of beverage codes linked at pre-tax and post-tax periods were created using a weighted average from Kantar purchase data at pre-tax and post-tax time periods. Another limitation is we missed the impact on reformulation between the announcement of the tax and when the taxed was implemented (the pre-implementation period) and thus underestimate reformulation. Furthermore, to the extent we did not carefully capture individual beverage heterogeneity within a wider brand-product specific categorization weighting approach using Kantar data, we might misrepresent some of the nuanced industry changes. We have added these two limitations more explicitly to the manuscript.

In summary, we find neither Spiteri nor Griffith addresses total intake, neither is based on actual consumption/intake, neither are used to evaluate real world changes total intake after a policy. They do offer more insight on the relative contributions of each component of reformulation—introduction of new products, changes in existing products, and discontinuing products—but our study goal was to examine changes in total intake after implementation of the South African SSB tax policy. In order to not overstate the novelty of our method, we have cited both the Spiteri and Griffith papers in our discussion, paragraph 5, mentioning their mathematical approach to disaggregating the components of reformulation on nutrient density, but still recognizing the difference in study aims.

**3.      Related the above point, how were new beverages introduced after the baseline treated in the analyses?**

Our response: Thank you for this comment. The data collection of nutrient information was a cross-sectional design, meaning any new products were included in the appropriate beverage codes. For example, a new, regular sugar Coca-Cola product with a sugar content above 4g/100mL that was reported in the post-tax period would have been included in the “Cold drink, carbonated, average (e.g. Cola, Cream Soda, Tonic)” beverage code, and the nutrient values of this beverage category were re-weighted according to the relative distribution of purchases in the Kantar data. A new diet product made with non-caloric sweeteners would have been placed in the “Cold drink, low-cal/artificially sweetened (e.g. Diet Cola)” beverage code.  **4.      It wasn't very clear to me how the nutritional values were attributed to beverages, in particular to what extent approximations had to be made using weights derived from purchase data. Could the authors provide more information on how detailed the beverage lists in the 24-hour recall was (i.e. how many beverage codes)? And how did the list of beverage codes in the 24-hour recall differ from the product level information available in Kantar? There is potentially some variability within beverage codes, which could affect the results.**

Our response: Thank you for this comment. There were 114 beverage codes that were categorized into Supplementary Table 1 beverage classification system based on their average sugar concentration, and whether this was above or below the 4g/100mL policy threshold.  **5.      Could you indicate when the tax was announced in the text and in the timeline. In the UK, there was for example an announcement effect which triggered reformulation before the introduction of the tax. As a result, the total effect of the tax might not be fully captured by the analyses presented in the paper if reformulation started before the baseline data collection. This might deserve to be discussed.**

Our response: Thank you for this comment about interpretation of our results. The initial announcement of a plan to tax sugary beverages was first given in February 2016, over two years before the SSB tax was ultimately implemented. We added a note for the announcement date for the plan to tax sugary beverages in paragraph 4 of the Discussion section. We interpret this to mean we could be potentially underestimating the reformulation effect if reformulation occurred after the announcement but before implementation, as found in the UK by Scarborough and colleagues in two papers.^1,2^ **6.      Could you explain a bit more on how the door to door sampling was conducted. Was there an element of randomisation for selecting the households to survey? Some important difference in socio-economic status were observed in the pre and post data collection waves. I was wondering whether the sampling method could have been responsible for part of those differences. You might have useful geographical location on the respondents at each wave, which could indicate systematic differences for example. Given the repeated cross-sectional study design, and the fact that few covariates are used in the models, I feel that I would need to be further reassured that the pre and post data collection do not systematically differ.**

Our response: Thank you for this comment. For the door to door survey, fieldworkers approached every household to ascertain if they met the entry requirement (aged 18-39 years) and if they were willing to participate. In households with 18-39 year olds, we selected one individual per household on a random basis. The household questionnaire was conducted digitally and included a geolocation. The geolocations provided us with useful maps to ensure that we covered all areas. We have added the note about geocodes to the manuscript, Data Sources and Measures section. A map from each data collection period is attached below to demonstrate. The areas where you can see single dots are the brick houses with the very dense dots are informal housing and flats. Open spaces are in general communal spaces such as playgrounds, churches, schools, police, health facility (clinic) or shops. There fewer of the lowest income areas sampled in the post-tax period, but we found no significant differences in calories from taxed or untaxed beverage intake according to socioeconomic status (LSM), likely because the total range of LSM in our sample is relatively narrow (zero high income households).

Pre-tax data collection geocodes

Post-tax data collection geocodes

**7.      I was wondering why other dietary information were not taken into account in the analyses. Could these for example provide an indication of the extent which the 2 samples systematically differ, which could then bias the results? Could these serve to adjust the results?**

Our response: Thank you for this comment. We considered adjusting for energy intake from foods (non-beverage calories), but adjusting for these could actually confound our results: given the before-and-after study design, we are interested in taxed, untaxed, and total changes in beverage intake. If there were any substitution effects between foods and beverages, these effects could be missed by adjusting for energy intake from foods. We added to the paper, “We did not adjust for intake from foods as we were interested in capturing the effects of the policy on beverage intake, which could include substitution effects.”
 **8.      Following the previous point, I was very curious to see a very large increase in the total volume consumed in the post-tax data collection in the adjusted model (+25%). How could this be explained? Was this also observed in the Kantar data analysed (paper under review cited)? Could this reflect a difference in socio-economic level between the 2 samples? Or rather the drought of 2018, as only briefly mentioned in the discussion. If the latter, this is potentially a massive confounding factor which should be further highlighted**

Our response: Thank you for this comment. As the reviewer notes, there was a large increase in total beverage volume intake in the post-tax period. The total beverage intake in the post-tax period was 223 mL greater than in the pre-tax period, and 177 mL of this increase was water.

When drought is added to the model, the results shift by less than a calorie when estimating energy intake from taxed beverages. A part of our sample data in the post period was collected when a drought was in function and strict water controls were enforced. When we controlled for those months, the results changed by only less than 1% for both taxed and untaxed beverages. This relates to the constant water scarcity in this and many other townships in South Africa. We noted these results in the Sensitivity Analysis section of Methods and Results sections.

Drought variable not adjusted for

Drought variable adjusted for

When estimating energy intake from untaxed beverages.

Drought variable not adjusted for

Drought variable adjusted for

**9.      Table 1 - could you indicate what the LSM category mean in the table**

Our response: LSM is a composite measure commonly used to segment based on socioeconomic status in South Africa. The relative ranking of each LSM level is publicly available on the South African Audience Research Foundation website: <http://www.saarf.co.za/lsm/lsm-presentations.asp> (February 2012 Presentation). To paragraph 2 below **Analytical Approach** in the Methods, we added a clarification on the meaning of the LSM range in our study, “With a total range from 1 to 10, the range of LSM in our study, from 3 to 6, includes low to middle socioeconomic status in South Africa.”
 **10.      Why LSM was the only socioeconomic indicator used? Were other indicators available?**

Our response: We have added to the second paragraph below **Analytical Approach** in the Methods: “LSM is a standardized composite measure commonly used to segment based on socioeconomic status in South Africa. It is based on 29 items, mostly including household assets (reference: <http://www.saarf.co.za/amps-technicalreport/technicalreport-Jan%202011%20-%20Dec%202011/data%20files/Technical/21%20-%20Tech%202011B%20~%20Pages%2092-97.pdf>). Other measures such as income are considered less reliable in South Africa for categorizing socioeconomic status.”

Although LSM was replaced by SEM in 2018 (<https://www.prc.za.com/wp-content/uploads/2017/08/New-SEM-socio-economic-segmentation-tool-explained.pdf>), it has been widely used by industry since 1989 (<http://www.saarf.co.za/lsm/lsms.asp>). We used LSM because we developed study material prior to 2018.
 **11.     It would be interesting to have an idea of the extent to which adjusted and unadjusted results differ. Maybe not necessarily in the main text.**

Our response: Thank you for this comment. We present the unadjusted versus adjusted results below. The differences are minor, actually. This may be due to the small heterogeneity within this township.

|  | **Unadjusted** | | **Adjusted** | |
| --- | --- | --- | --- | --- |
| **Taxed** | **Pre-tax Mean  (95% CI)** | **Post-tax Mean  (95% CI)** | **Pre-tax Estimate  (95% CI)** | **Post-tax Estimate (95% CI)** |
| Sugar (g) | 28.4 (27.0 to 29.7) | 19.8  (18.5 to 21.1) | 28.8  (27.3 to 30.4) | 19.8  (18.5 to 21.1) |
| Energy (kcal) | 119  (113 to 125) | 82  (76 to 87) | 121  (114 to 127) | 82  (76 to 87) |
| Volume (mL) | 308  (293 to 323) | 198  (186 to 211) | 315  (297 to 332) | 198  (185 to 211) |
| **Untaxed** |  |  |  |  |
| Sugar (g) | 15.0  (14.1 to 15.9) | 20.2  (19.1 to 21.2) | 15.0  (13.9 to 16.0) | 20.3  (19.2 to 21.4) |
| Energy (kcal) | 106  (100 – 112) | 133  (127 to 139) | 105  (99 to 112) | 135  (128 to 141) |
| Volume (mL) | 588  (565 to 610) | 929  (905 to 954) | 587  (563 to 610) | 926  (899 to 953) |

**12.     Page 3 line 94: "while these data are important, they exclude many sources of SSBs which dietary measures overcome". Could you give an example of those sources of SSBs.**

Our response: Thank you for this comment. All measures have their strengths and weaknesses but when wanting to link to the individual level only dietary data makes that possible. We have added explanation to this claim: “Previous work estimated crude store-intercepts and SSB frequency questionnaires for small city evaluations, but these methods miss other sources of consumption and do not represent key segments of a country ^3–7^. Others have used high quality household purchase data or cruder aggregate sales data ^8–11^. While these data are important, they exclude many sources of SSBs which dietary measures overcome. For example, collecting purchase information through exit interviews at stores could miss other locations where participants shopped. Food purchase studies can also miss incidental beverage purchases and underestimate the total consumption. Another source of consumption data, aggregate sales from Euromonitor International can miss the sales from vending and smaller nonchain stores. In contrast, individual dietary intake data record what each individual actually consumes, regardless of where it was purchased. Although 24-hour recall data are susceptible to both random and systematic errors, they are considered the least biased self-report dietary intake instruments and the most appropriate for evaluating the effects of an intervention by the National Cancer Institute^12^.”

**13.     What distributional assumptions are used for the second part of the model (for which a 'log-link' function is specified)? Do the data meet the assumptions?**

Our response: Thank you for this comment. The gamma distribution with a log-link is appropriate for data that are continuous, positive, and right-skew, a common distribution for dietary intake data as beverage intakes have no upper limit. Our data are also continuous, positive, and right-skewed. We added this detail to the paper in *Estimation of Beverage Intake* in the Analytical Approach of the Methods.
 **14.     About the 2-part model: the type of model seems to make sense for the analysis of some food groups, which are rarely consumed. However, this might be less the case for total beverage volumes or sugar, or even reformulated products which contain a mixture of sugar and artificial sweeteners. I was wondering if the authors could reassure the reader that the results are not affected by this methodological choice, by reproducing some of the more general results using an alternative modelling strategy, where the 2 part-model does not feel as much justified.**

Our response: Thank you for this important methodological comment. The reviewer is correct that the two-part model has an advantage in analyzing data with many zero observations (i.e. few consumers of a beverage type). However, it does not conversely have drawbacks if it is used in a category with high consumers (convention typically would be 90% or more consumers). For example, there would be a high probability to consume total (any) beverages, so the output of the model would be very similar to the second part of the model: the consumption day amount. We tested this possibility, finding the following results for total beverages:

Estimating kcal from total beverages using two part model:

Estimating kcal from total beverages using generalized linear model with log link only:

Following the reviewer’s advice, we’ve added this to our sensitivity analysis section of the Methods: **“**We tested whether the two-part model was less appropriate for modeling total beverage intake, with a percentage of consumers over 90%. We found less than a 1 kcal difference between results using the two-part model and solely a generalized linear model with a log-link, which would have no effect on the reported results.**”**

**15.     Could the authors provide all the coefficients of the fitted model for peer review purposes, and not just the adjusted predicted mean of the outcome.**

Our response: Thank you for this comment, we have provided the coefficients in tables, added as a supplemental file: Model Coefficients.

**16.     Sensitivity analysis. No change in energy intake when moving LSM from the analysis is no indication of an absence of bias, as both intake values could be biased indeed. To rule out whether results could be biased, the authors should assess whether missingness on LSM depends on the outcome (intake), conditional on the covariates (see e.g. Carpenter and Kenward, Multiple Imputation and its applications)**

Our response: Thank you for this comment. It can also be due to the lack of major variability in LSM score. We concur it is correct to assess whether missingness on LSM depends on the outcome (beverage intake). To answer this question, we conducted a logistic regression model, setting missingness on LSM (misslsm==1 if LSM is missing), with the following results:

For taxed beverages

For untaxed beverages

The odds of being missing are not statistically significant at the p<0.05 level for taxed nor untaxed beverages. Although sex is associated, it is controlled for in the models, and sex was evenly distributed between both phases. We have added this sensitivity analysis to the end of the Methods section and removed the previous one, which the reviewer rightly suggests does not correctly indicate an absence of bias.

We added to the final section of the Results, Sensitivity Analyses, “We found missingness on LSM did not depend on the outcome (beverage intake). This suggests our intake estimates are not biased due to the missing LSM data.”

**17.     Page 8 line 325-326: could the authors avoid the word 'statistically significant', this only makes sense with reference to a certain level of significance. Also, the sentence seems to indicate that there was strong evidence of energy reduction overall, whereas the 95%CI includes 0, and the p-value (which is not reported) appears to be higher than 0.01.**

Our response: Thank you for this comment. We removed the term “statistically significant” from the sentence and removed the mention of total energy from beverages, leaving only the result for reduction in total sugar intake from beverages, including the p value (p=0.004) instead of the term “statistically significant.”

**18.     Page 5 line 170 "our key outcome variables include mean adjusted" - I am not sure what the authors mean by this. Outcomes (i.e. variables) are intake, not "mean adjusted" intake (i.e. summary statistic)?**

Our response: Thank you for this comment. This was a typographical error and did not belong. We have removed the words “mean adjusted,” and the sentence now reads, “Our key outcome variables include intake of total sugar…”
 **19.     Figures 2-4. From the caption and figures it is not very clear what behavioural, and reformulation mean. I would be more explicit so that the figure can be read independently.**

Our response: Thank you for this comment. We have changed the x-axis labels to “Post-Tax: Behavior Only” and “Post-Tax: Reformulation included” to clarify that both of these columns are from post-tax data. We have also added more detail to the figure captions to clarify what Behavior Only and Reformulation Included mean: “Post-Tax: Behavioral Only refers to estimates of beverage intake using only the pre-tax FCT, which keeps nutrient content constant. Post-Tax: Reformulation Included refers to estimates of beverage intake using the post-tax FCT, which accounts for reformulation changes in nutrient content of beverages.”

**20.     Why didn't you run the analysis on households who were interviewed at each wave as a sensitivity analysis, as opposed to running it on those who were not. Focusing on the 'longitudinal sample' would ensure that the population is the same, which would rule out the concern that the populations are different between the 2 waves.**

Our response: Thank you for this comment. We are not able to call our sample “longitudinal” because of the cross-sectional design, and the only information we have at post-tax is whether the respondent was part of the previous data collection. This means we know which records are repeats in the post-tax period, but they cannot be linked to records in the pre-tax period (no repeats possible in initial data collection). Although we are not able to test the longitudinal sample, we see almost no differences in results whether or not we control for the only category along which the sample differed—LSM—when restricted to the common sample.

Estimated calories from taxed beverages including LSM in the model:

Pre-tax: 120.9 (95% CI 114.2 – 127.5) kcal/capita/day

Post-tax: 81.6 (95% CI 76.1 – 87.2) kcal/capita/day

Estimated calories from taxed beverages without LSM in the model:

Pre-tax: 121.0 (95% CI 114.7 – 127.4) kcal/capita/day

Post-tax: 81.7 (95% CI 76.2 – 87.2) kcal/capita/day **Reviewer #3: This is a well-conducted study on beverage consumptions by young adults in Langa, South Africa before and after a national sugar-sweetened beverage tax. The study design, datasets, statistical methods and analyses, and presentation of results (tables and figures) are mostly adequate. However, there are a few issues especially interpretation of results needing attention.

1) The title and the abstract. The title is "Taxed and untaxed beverage consumption by...", however in the abstract, it's all about taxed beverage consumption in the findings and conclusion and nothing about untaxed and total beverage consumption. It's an incomplete summary/finding which doesn't match the title.**

Our response: Thank you for this comment. We have revised the abstract to present more results including taxed, untaxed, and total beverages.
 **2) Interpretation of study results. Table 2 is a key table summarising the main findings of the study. We can see clearly that after introducing the SSB tax there were decreases in sugar, energy and volume consumption in taxed beverage but increases in untaxed beverage, and overall slight or no change in total beverage consumption. So far the paper is mainly focused on the results of taxed beverage consumption changes but largely ignored the untaxed and total beverage results, which is inadequate. To evaluate the effect of SSB tax fairly a holistic approach of presenting and interpreting all the results including taxed, untaxed and total beverage consumptions is needed in order to provide a complete picture of the effectiveness and impact of SSB tax policy. Balanced presentation and interpretation of results are needed in discussion, conclusion and in abstract.**

Our response: Thank you for this comment. We do concur with you that the total net impact, including all substitutes and complementary changes, is what counts for any tax that is being evaluated. We chose to focus on taxed beverage shifts to maintain consistencies with previous SSB tax evaluations to date.^1–5,7,11^ However, we have also revised the Abstract, Discussion, and Conclusion to also discuss the untaxed and total results, thereby presenting a more balanced view of the study results.  **3) The two-part model is key in estimation of beverage intake. Authors said the reason why the model was used is in the first part to account for beverage groups that have a high percentage of non-consumers (likelihood of consumption). Can authors make it explicit on what 'non-consumers' means? Were these young people consumer or non-consumer? What's the percentage and how it was modelled? Unless all these are made clear, readers will have doubts on the validity of using this two-part model.**

Our response: The two part model is useful for modeling beverage intakes that have high percentage of non-consumers (typically considered when 10% or more of the study sample did not consume the beverage). We added a brief explanation of non-consumers in lines 271-4: “We estimated beverage intake from 24-h recalls using a two-part model implemented with the Stata twopm command to account for beverage groups that have a high percentage of non-consumers, meaning study participants who did not report a particular beverage category consumed.” Participants can therefore be consumers of some beverages but non-consumers of the exact beverage category we are analyzing. The two part model in this study consists first of a binary choice model that accounts for the probability to consume, followed by a generalized linear model to estimate the consumption day amount. This means that if a beverage category has on the other hand a very high proportion of consumers (over 90% of the sample consumed a beverage in that category), the probability to consume is very high, and the results from the model are driven by the second component of the model, the consumption-day amount.

We have added to the Sensitivity Analysis section of the Methods and Results to assure readers there was no problem using the two-part model for beverage categories with a high percentage of consumers (lines 457-9): “We tested whether the two-part model was less appropriate for modeling total beverage intake, with a percentage of consumers over 90%. We found less than a 1 kcal difference between results using the two-part model and solely a generalized linear model with a log-link.”

**References used in this document**

1.	Scarborough, P. *et al.* Impact of the announcement and implementation of the UK Soft Drinks Industry Levy on sugar content, price, product size and number of available soft drinks in the UK, 2015-19: A controlled interrupted time series analysis. *PLoS Med.* 1–19 (2020). doi:https://doi.org/10.1371/journal.pmed.1003025

2.	Bandy, L. K., Scarborough, P., Harrington, R. A., Rayner, M. & Jebb, S. A. Reductions in sugar sales from soft drinks in the UK from 2015 to 2018. *BMC Med.* **18**, 1–10 (2020).

3.	Cawley, J., Frisvold, D., Hill, A. & Jones, D. The impact of the Philadelphia beverage tax on purchases and consumption by adults and children. *J. Health Econ.* **67**, 102225 (2019).

4.	Fletcher, J. M., Frisvold, D. & Tefft, N. Taxing soft drinks and restricting access to vending machines to curb child obesity. *Health Aff.* **29**, 1059–1066 (2010).

5.	Lee, M. M. *et al.* Sugar-Sweetened Beverage Consumption 3 Years After the Berkeley, California, Sugar-Sweetened Beverage Tax. *Am. J. Public Health* e1–e3 (2019). doi:10.2105/AJPH.2019.304971

6.	Falbe, J. *et al.* Impact of the Berkeley Excise Tax on Sugar-Sweetened Beverage Consumption. *Am. J. Public Health* **106**, e1–e7 (2016).

7.	Zhong, Y., Auchincloss, A. H., Lee, B. K. & Kanter, G. P. The Short-Term Impacts of the Philadelphia Beverage Tax on Beverage Consumption. *Am. J. Prev. Med.* **55**, 26–34 (2018).

8.	Arantxa Colchero, M., Rivera-Dommarco, J., Popkin, B. M. & Ng, S. W. In Mexico, evidence of sustained consumer response two years after implementing a sugar-sweetened beverage tax. *Health Aff.* **36**, 564–571 (2017).

9.	Caro, J. C. *et al.* Chile’s 2014 sugar-sweetened beverage tax and changes in prices and purchases of sugar-sweetened beverages: An observational study in an urban environment. *PLoS Med.* **15**, 1–19 (2018).

10.	Silver, L. D. *et al.* Changes in prices, sales, consumer spending, and beverage consumption one year after a tax on sugar-sweetened beverages in Berkeley, California, US: A before-and-after study. *PLoS Med.* 1–19 (2017). doi:10.1371/journal.pmed.1002283

11.	Fletcher, J. M., Frisvold, D. E. & Tefft, N. The effects of soft drink taxes on child and adolescent consumption and weight outcomes. *J. Public Econ.* **94**, 967–974 (2010).

12.	Thompson, F. E. *et al.* The National Cancer Institute’s Dietary Assessment Primer: A Resource for Diet Research. *J Acad Nutr Diet* **115**, 1986–1995 (2015).
