# Supplementary material for: Taxed and untaxed beverage intake by South African young adults after a national sugar-sweetened beverage tax: A before-and-after study
Source: PLoS Med. 2021 May 25;18(5):e1003574. doi: 10.1371/journal.pmed.1003574 (PMC8148332; doi:10.1371/journal.pmed.1003574)
Supplement: Attachment — Submitted filename: PMEDICINE-D-20-03807R2 Response document_final.docx [file pmed.1003574.s010.docx]

PMEDICINE-D-20-03807R2 Response to Editor and Reviewer Comments

Dear editors and reviewers, thank you for your helpful comments. We are happy that the manuscript is strengthened and more suitable for publication. A point-by-point description of our responses and changes to the manuscript can be found below. We have also included in this document responses to two requests regarding (1) funding and (2) data availability after our Feb 11 submission.

We look forward to your further review.

Michael Essman on behalf of co-authors

Thank you for addressing the previously stated issues. Before we proceed, please address the following issues:

Thank you for providing a Financial Disclosure statement in the submission form. This statement is published with the article, therefore should be completed in full sentences and contain the exact wording you wish to be published.

i) Please ensure that the funders and grant numbers match between the Financial Disclosure field and the Funding Information tab in your submission form. Note that the funders must be provided in the same order in both places as well.

**Our response: Thank you for this correction. The Financial Disclosure statement reads:**

**Funding for this study comes from Bloomberg Philanthropies (**[**https://www.bloomberg.org/**](https://www.bloomberg.org/)**; received by BP), including Subcontract #5108311 with the University of the Western Cape. This research also received support from the Population Research Infrastructure Program awarded to the Carolina Population Center (P2C HD050924) at The University of North Carolina at Chapel Hill by the Eunice Kennedy Shriver National Institute of Child Health and Human Development. ME was supported by a Predoctoral Fellowship from NIH Training Grant T32 HL129969. Other support includes student scholarships from International Development Research Center (IDRC) Project number 108425-001 and the DST/NRF Centre of Excellence in Food Security project number 180401 (https://www.uwc.ac.za/Faculties/CHS/soph/Pages/The%20Centre-of-Excellence-in-Food-Security.aspx). The funders had no role in study design, data collection and analysis, decision to publish, or preparation of the manuscript.**

**This statement matches—both in number and order—the information entered into the Funding Information tab.**

Please note that the contact for accessing your data cannot be an author on the manuscript. Please therefore replace the current contact information with a 3rd party contact, such as an institutional data access committee.

**Our response: Thank you for this comment. We are uploading the data to a third party location, the Carolina Digital Repository (**[**https://cdr.lib.unc.edu/**](https://cdr.lib.unc.edu/)**). The contacts for data requests are Donna Miles (**[**drmiles@email.unc.edu**](mailto:drmiles@email.unc.edu)**), Director of Research Programming Services at the UNC Carolina Population Center, and Jessica Ostrowski (**[**jessica.ostrowski@unc.edu**](mailto:jessica.ostrowski@unc.edu)**).**

**The Data Availability statement now reads:**

**Data analyzed for this paper form part of a primary project which is currently being written up in other publications. Select relevant data used for this paper will therefore only be available upon request and only granted for replication purposes. Data are available from the UNC Carolina Digital Repository (**[**https://cdr.lib.unc.edu/**](https://cdr.lib.unc.edu/)**). For data inquiries, please contact Donna Miles (**[**drmiles@email.unc.edu**](mailto:drmiles@email.unc.edu)**) and Jessica Ostrowski (**[**jessica.ostrowski@unc.edu**](mailto:jessica.ostrowski@unc.edu)**).**

• Please revise your title according to PLOS Medicine's style. Your title must be nondeclarative. For example, “Taxed and untaxed beverage intake by South African young adults after a national sugar-sweetened beverage tax: a before-and-after study.”

**Our response: Thank you for this correction. We have revised the title to, “Taxed and untaxed beverage intake by South African young adults after a national sugar-sweetened beverage tax: a before-and-after study” to be nondeclarative.**

• Under the Competing Interests statement, please declare that Barry Popkin is an Academic Editor with PLOS Med.

**Our response: Thank you for this comment. The Competing Interest Statement now reads, “We have read and understood PLOS Medicine’s policy on declaration of interests and ME, LST, TF, SWN, and ECS declare that they have no competing interests. BP is on the PLOS Medicine editorial board and otherwise has no competing interests.”**

• In the Author Summary, please avoid assertions of primacy. Specifically, the second bullet point under “Why was this study done?”, use the phrase, “To our knowledge, this is the first…”

**Our response: Thank you for this comment. We have updated the second bullet point under “Why was this study done?”** **to, “To our knowledge, this is the first study to examine the effects of an SSB tax using detailed 24-hour recall dietary intake data focused on a low-income population from the Global South.”**

**We also updated the third bullet point under “Why was this study done?” to, “To our knowledge, this is the first study to evaluate real world changes from an SSB tax policy by empirically quantifying how much of the overall change in sugar from taxed beverages came from consumers’ behavioral changes versus reformulation of beverages.”**

**We also updated the third paragraph of the Introduction to (Line 157), “To our knowledge, it is also the first SSB tax evaluation of a national tax to use detailed 24-hour recall dietary data.”**

• Your study is observational and therefore causality cannot be inferred. Please remove language that implies causality throughout the manuscript, such as “The majority of this increase was due to ...” Refer to associations instead. For example, in the third and fourth bullet point under “What did the researchers do and find?”, line 359, line 378 to 390, line 393, Discussion section, and so forth.

**Our response: Thank you for this comment. We changed language implying causality to language describing pre-post differences and outline the many changes below. As a general point, we removed language implying causality related to effects of the HPL, which is ultimately the concern of this study design and the critique regarding causality. However, we do not remove every instance of phrases such as “increase” or “decrease” as these are objective descriptions of the differences in the data, and do not necessarily entail causal claims about the effects of the HPL. The causal question relates to whether the HPL was the cause of these changes—which occurred no matter the cause. As long as we are not claiming the HPL was the cause of all of our effects, we are on solid ground describing our results as increases or decreases. For such a before-and-after study, measured outcomes can increase or decrease in the post-tax period without implying the event (HPL implementation) was the cause of those differences.**

**In response to the critique, we tempered the causal language in several areas. We outline each of the key changes below (Line numbers refer to latest submitted manuscript to avoid confusion and keep all numbers up to date):**

- **Third bullet point under “What did the researchers do and find?”:**
  - **Original: “The majority of this increase (52%) was due to increased water consumption”**
  - **Edited to: “Water accounted for the majority (52%) of the pre-post difference in volume of untaxed beverage intake.”**
    - **Note from authors: The other content of the third bullet point merely refers to decreases, which give a direction for differences, and are not inherently causal.**
- **Fourth bullet point under “What did the researchers do and find?”:**
  - **Original: Of the 9.0 g per capita per day (31%) total reduction in sugar from taxed beverages, 6.4 g per capita per day (22%) was due to behavioral change, and an additional 2.6 g per capita per day (9%) reduction was due to reformulation.**
  - **Edited to: Of the 9.0 g per capita per day (31%) total reduction in sugar from taxed beverages, 6.4 g per capita per day (22%) was due to behavioral differences. When accounting for reformulation, there was an additional 2.6 g per capita per day (9%) reduction in the post-tax period.**
    - **Note from authors: differences in behavior (recorded intake) at two time points.**
- **Line 371**
  - **Original: “The majority of this increase (52%) was due to increased water consumption”**
  - **Edited to (Line 368 revised manuscript): “water accounted for the majority (52%) of this pre-post difference in untaxed beverage intake.”**
- **Line 400 (entire paragraph):**
  - **Original: We found a stepwise reduction in sugar, energy, and volume from taxed beverages from pre-tax to post-tax with the pre-tax FCT, which isolates behavioral changes with no reformulation, and greater reductions using the updated post-tax FCT, which captures reformulation’s marginal effect (Figs. 2-4). For sugar, we estimated a change from 28.8 g/capita/day (95% CI 27.3 to 30.4) to 22.4 (95% CI 21.1 to 23.8), a 22.2% reduction, due to behavioral change and an additional 9.2% reduction to 19.8 g/capita/day (95% CI 18.5 to 21.1) due to reformulation (Fig. 2). For energy, we estimated a change from 121 kcal/capita/day (95% CI 114 to 127) to 92 (95% CI 86 to 97), a 24.1% reduction, due to behavioral change and an additional 8.4% reduction to 82 kcal/capita/day (95% CI 76 to 87) due to reformulation (Fig. 3). For volume, we estimated a change from 315 mL/capita/day (95% CI 297 to 352) to 241 (95% CI 227 to 256), a 23.3% reduction, due to behavioral change and an additional 13.7% reduction to 198 mL/capita/day (95% CI 185 to 211) due to reformulation (Fig. 4).**
  - **Edited to: For taxed beverages, we found greater pre-post differences when accounting for reformulation. Sugar intake from beverages** **was 28.8 g/capita/day (95% CI 27.3 to 30.4) in the pre-tax period and 22.4 (95% CI 21.1 to 23.8) in the post-tax period (-22.2%). When accounting for reformulation, there was a 9.2% additional reduction to 19.8 g/capita/day (95% CI 18.5 to 21.1) in the post-tax period compared to the pre-tax period (Fig. 2). Energy intake was 121 kcal/capita/day (95% CI 114 to 127) in the pre-tax period and 92 (95% CI 86 to 97) in the post-tax period (-24.1%). When accounting for reformulation, there was an 8.4% additional reduction to 82 kcal/capita/day (95% CI 76 to 87) in the post-tax period compared to the pre-tax period (Fig. 3). Volume intake was 315 mL/capita/day (95% CI 297 to 352) in the pre-tax period and 241 (95% CI 227 to 256) in the post-tax period (-23.3%). When accounting for reformulation, there was a 13.7% additional reduction to 198 mL/capita/day (95% CI 185 to 211) in the post-tax period compared to the pre-tax period (Fig. 4).**
- **Line 413:**
  - **Original: For untaxed beverages, energy increased 33.9% from 105 kcal/capita/day (95% CI 99 to 112) to 141 (95% CI 134 to 148), but reformulation attenuated this increase to 135 (+27.5%) kcal/capita/day (95% CI 76 to 87) (Fig. 3).**
  - **Edited to: For untaxed beverages, energy intake was 105 kcal/capita/day (95% CI 99 to 112) in the pre-tax period and 141 (95% CI 134 to 148) in the post-tax period (+33.9%). When accounting for reformulation, there was less of an increase to 135 (+27.5%) kcal/capita/day (95% CI 76 to 87) in the post-tax period (Fig. 3).**
- **Line 420 entire paragraph:**
  - **Original: For total beverages, we estimated a 3.1% reduction in sugar from 43.8 g/capita/day (41.9 to 45.7) to 42.4 (40.9 to 44.0) due to behavioral change and an additional 5.4% reduction to 40.1 (95% CI 38.5 to 41.6) g/capita/day due to reformulation, making the total difference from pre-tax statistically significant (p=0.004) (Fig. 2). Energy from total beverages increased from 226 kcal/capita/day (95% CI 217 to 235) to 233 (95% CI 224 to 241) accounting for behavioral change only, but decreased to 216 (-4.6%) kcal/capita/day (95% CI 208 to 87) accounting for reformulation effects, although this reduction was not statistically significant (Fig. 3).**
  - **Edited to: For total beverages, sugar intake was 43.8 g/capita/day (41.9 to 45.7) in the pre-tax period and 42.4 (40.9 to 44.0) in the post-tax period (-3.2%). When accounting for reformulation, there was a 5.4% additional reduction to 40.1 (95% CI 38.5 to 41.6) g/capita/day in the post-tax period, making the total difference from pre-tax statistically significant (p=0.004) (Fig. 2). Energy from total beverages was 226 kcal/capita/day (95% CI 217 to 235) in the pre-tax period and 233 (95% CI 224 to 241) in the post-tax period. When accounting for reformulation, there was a 4.4% total reduction to 216 kcal/capita/day (95% CI 208 to 224) in the post-tax period, although this reduction was not statistically significant (Fig. 3).**
- **Discussion section (paragraph 1 Line 507)**
  - **Original: Our results indicate overall that behavior change of these adults was responsible for reductions of 22% of taxed beverage sugar intake and 3.2% of total beverage sugar intake compared to pre-tax levels, and our estimate of reformulation was the remainder of the post-tax impact.**
  - **Edited to: Our results indicate overall that behavior change of these adults was associated with reductions of 22% in taxed beverage sugar intake and 3.2% in total beverage sugar intake compared to pre-tax levels, and our estimate of reformulation was the remainder of the post-tax difference.**
    - **Note from authors: we do not consider this statement about reformulation to be a causal claim about the effect of the tax, it is simply the meaning of the updated FCT approach that accounts for reformulation.**
- **Discussion section (paragraph 6)**
  - **Original: Water was a major driver, accounting for 177 mL/capita/day (52%) of the increase in untaxed beverage volume (S3 Table).**
  - **Edited to: Water was a major component, accounting for 177 mL/capita/day (52%) of the increase in untaxed beverage volume (S3 Table).**
- **Discussion section (Line 585)**
  - **Original: The magnitude of the HPL effect on taxed beverage consumption in this study may not be generalizable to higher income populations, given that other studies have found the largest changes in sugary beverage intake among the lowest income groups analyzed.**
  - **Edited to: The magnitude of changes in taxed beverage consumption after the HPL in this study may not be generalizable to higher income populations, given that other studies have found the largest changes in sugary beverage intake among the lowest income groups analyzed.**
- **Discussion section (Line 588)**
  - **Original: However, the methods used in this study are useful for future work as they can separate the two crucial effects of behavioral change and reformulation regardless of the total effect of a tax on income groups.**
  - **Edited to: However, the methods used in this study are useful for future work as they can separate the two crucial components of behavioral change and reformulation regardless of the total effect of a tax on income groups.**
- **Conclusion (Line 619)**
  - **Original: Using a large sample of a high-consuming, low-income population, we found large reductions in taxed beverage intake, separating the effects of behavioral change from reformulation.**
  - **Edited to: Using a large sample of a high-consuming, low-income population, we found large reductions in taxed beverage intake, separating the components of behavioral change from reformulation.**

• Please attenuate you statement in line 539 to 540.

**Our response: Thank you for this comment. The original sentence reads (Line 590 of revised), “Finally, although we are able to separate the effects of behavioral change on beverage consumption from reformulation effects, we cannot isolate the specific types of reformulation effects that may have occurred.” We have modified this sentence to, “Finally, although we examined pre-post differences in beverage intakes under two scenarios, first accounting for differences in intake only, followed by differences in intake plus reformulation effects, we cannot isolate the specific types of reformulation that may have occurred, nor identify any causal effects.”**

• Change Methods, line 3 to rename Appendix A as Supporting Information file A or 1 and so forth.

**Our response: Thank you for this comment. We changed the Appendix A study protocol name to “S1 Protocol” and have uploaded the file as “S1 Protocol. Study Protocol.” We also renamed our Supplementary Tables in the appropriate Supporting Information format: S1 Table, S2 Table, S3 Table, S4 Table, and S5 Table.**

• In Table 2 and 5, please provide exact p-values unless p <0.001

**Our response: Thank you for this comment. We have provided exact p-values in these tables, except when p <0.001. For Table 2, we added the following note: “Exact p-values are reported unless p <0.001.” For Supplementary Table 5, we added the following note: “P-values relate to within-row comparisons (post-tax compared to pre-tax). Exact p-values are reported unless p <0.001.”**

• In the tables, please explain, in the legend, what the error bars represent

**Our response: Thank you for this comment. We added, “Values in parentheses represent 95% Confidence Intervals (CI)” to the legend of Table 2 and Supplementary Tables 2-5. We changed the format of Supplementary Tables 2-4 to more consistently resemble that of Table 2: “value (95% CI)” rather than separating value, lower bound, upper bound.**

• Do not underline the text in line 445-446

**Our response: Thank you for this comment. We have removed all underlining.**

• Please add the following statement, or similar, to the Methods: "This study is reported as per the Strengthening the Reporting of Observational Studies in Epidemiology (STROBE) guideline (S1 Checklist)."

**Our response: Thank you for this comment. We have added the suggested statement exactly as written to the first paragraph of the Methods section.**

• Under references, please indicate the access date for all websites consistently.

**Our response: Thank you for this comment. For all websites, we include the phrase “cited YYYY MO DD.” We also removed the electronic tag [Internet] for regular journal articles (not websites). This was a default function of the reference manager software that we disabled.**

• Please update reference 42 – it is no longer under review.

**Our response: Thank you for this comment. Following the** [**National Library of Medicine Guidelines**](https://www.ncbi.nlm.nih.gov/books/NBK7240/#A42793) **for articles that have been accepted for publication but are forthcoming, we updated the reference to:**

**Stacey N, Edoka I, Hofman K, Swart R, Popkin BM, Ng S. Changes in Beverage Purchases Following the Announcement and Implementation of South Africa’s Health Promotion Levy: An Observational Study. Lancet Planet Heal. Forthcoming 2021.**

• Indicate that Supporting Information file “Diet final” is a survey. Consider renaming it as such.

**Our response: We renamed this Supporting Information file “Diet Survey.”**

• In the third bullet point of 'What did the researchers find?' there is a typo, where 'non-taxed beverages' are referred to as 'taxed beverages.’ Please revise this.

**Our response: Thank you for spotting this typo. We have fixed the sentence in the third bullet point of the ‘What Did the Researchers Do and Find?’ section:**

- **The intake of sugar, calories and volume of untaxed beverages increased by 5.3 g (36%), 30 kcal (29%), and 339 mL (58%) per capita per day, respectively.**

------------------------------------------------------------
Requests from Editors:

Comments from Reviewers:

Reviewer #1: The authors have fully addressed all of my concerns at the first review round and I have only one very small further comment to make. In the third bullet point of 'What did the researchers find?' there is a typo, where 'non-taxed beverages' are referred to as 'taxed beverages'.

Thank you for the opportunity to review this paper.

**Our response: Thank you for spotting this typo. We have fixed the sentence in the third bullet point of the ‘What Did the Researchers Do and Find?’ section:**

- **The intake of sugar, calories and volume of untaxed beverages increased by 5.3 g (36%), 30 kcal (29%), and 339 mL (58%) per capita per day, respectively.**

**Thank you for your comments that helped us strengthen the manuscript.**

Reviewer #2: It was a pleasure to read the revised version of the manuscript. The authors have done an excellent job in systematically addressing all my initial comments and concerns, and I have no further comments.
The paper looks stronger in my view and will be a nice contribution to the journal.

**Our response: Thank you for your comments that helped us strengthen the manuscript.**

Reviewer #3: Thanks authors for their great effort to improve the manuscript. I am satisfied with the response and revision. No further issues needing attention.

**Our response: Thank you for your comments that helped us strengthen the manuscript.**
